# Identification of Combinations of Plasma lncRNAs and mRNAs as Potential Biomarkers for Precursor Lesions and Early Gastric Cancer

**DOI:** 10.1155/2022/1458320

**Published:** 2022-02-11

**Authors:** Lu Chen, Changhui Ge, Xiuxue Feng, Hanjiang Fu, Shasha Wang, Jie Zhu, Enqiang Linghu, Xiaofei Zheng

**Affiliations:** ^1^Department of Experimental Hematology and Biochemistry, Beijing Key Laboratory for Radiobiology, Beijing Institute of Radiation Medicine, Beijing 100850, China; ^2^Division of Gastroenterology, The First Medical Center of Chinese PLA General Hospital, Beijing 1000853, China

## Abstract

Patients with gastric cancer (GC) are usually first diagnosed at an advanced stage due to the absence of obvious symptoms at an early GC (EGC) stage. Therefore, it is necessary to identify an effective screening method to detect precursor lesions of GC (PLGC) and EGC to increase the 5-year survival rate of patients. Cell-free RNA, as a biomarker, has shown potential in early diagnosis, personalised treatment, and prognosis of cancer. In this study, six RNAs (CEBPA-AS1, INHBA-AS1, AK001058, UCA1, PPBP, and RGS18) were analysed via real-time quantitative polymerase chain reaction (RT-qPCR) using the plasma of patients with EGC and PLGC to identify diagnostic biomarkers. The receiver operating characteristic (ROC) curve analysis was used to evaluate the diagnostic accuracy. Among the six RNAs, four lncRNAs (CEBPA-AS1, INHBA-AS1, AK001058, and UCA1) were upregulated and two mRNAs (PPBP and RGS18) were downregulated in the plasma of patients with PLGC and EGC. According to the findings of the ROC analysis, the four-RNA combination of INHBA-AS1, AK001058, UCA1, and RGS18 had the highest area under the curve (AUC) value for determining risk of GC in patients with PLGC and the six-RNA combination including CEBPA-AS1, INHBA-AS1, AK001058, UCA1, PPBP, and RGS18 had the highest AUC value for determining the risk of GC in patients with EGC. The results suggest the potential usefulness of noninvasive biomarkers for the molecular diagnosis of GC at earlier stages.

## 1. Introduction

Gastric cancer (GC) is the fifth most common malignant tumour and the fourth leading cause of cancer-related death globally. According to the World Health Organization's global cancer data, 1.09 million new cases and 770,000 deaths occurred due to GC in 2020 [1]. Due to the lack of awareness of early screening for tumours, lack of obvious symptoms in precancer and early cancer stages, and unavailability of more advanced means of early tumour screening, most patients get diagnosed after progressing to advanced stage GC. A survey shows that the 5-year survival rate of patients with early GC (EGC) is >90%, while that of patients with advanced GC is only approximately 30% [2]. Therefore, attention must be paid to the timely detection and treatment of EGC.

Although gastroscopy combined with tissue biopsy is the “gold standard” method for tumour detection and diagnosis, endoscopy is limited by the type of equipment and physician techniques and the results vary [3]. Furthermore, because it is not possible to distinguish the heterogeneity of a tumour using tissue specimens, discrepancies often occur between gastroscopic results and pathological diagnosis [4]. Several noninvasive detection methods for GC are available, including circulatory biomarkers. However, currently known tumour antigens in serum, including carcinoembryonic antigen (CEA), carbohydrate antigen 19-9 (CA19-9), CA72-4, and pepsinogen [5,6], are of little use in GC diagnosis due to their low specificity and sensitivity [7]. Thus, identifying additional biomarkers is urgently needed to improve the diagnostic efficiency of EGC.

Recent studies have highlighted the potential use of circulating cell-free RNA (cfRNA), such as mRNA, microRNA (miRNA), and long noncoding RNA (lncRNA), in the peripheral blood for cancer diagnosis, prognosis, and monitoring of response to anticancer therapy [8]. Previous studies indicated that cfRNA could originate from necrotic or apoptotic tumour cells [9,10] or be actively released by tumour cells in microvesicles [11]. cfRNAs obtained from the plasma and serum as biomarkers for detecting and diagnosing EGC [12,13], such as lncRNA PCGEM1 and UCA1 [14,15]; circRNA circ_0141633 and circ_0004771 [16,17]; miRNA miR-21 and miR-26a [18,19]; mRNA SLC6A3 and L1CAM [20,21]; and combinations of lncRNAs [22], miRNAs [23], and mRNAs [24].

In the present study, we investigated the expression of four lncRNAs and two mRNAs in the plasma of patients with EGC and precursor lesions of GC (PLGC) using real-time quantitative polymerase chain reaction (RT-qPCR). Furthermore, the specificity and sensitivity of single and combination RNAs were evaluated for the diagnosis of EGC and PLGC. Our study indicated that the combination of plasma lncRNA and mRNA expression might be a useful biomarker for GC diagnosis.

## 2. Materials and Methods

### 2.1. Plasma Samples

Plasma samples from healthy participants (*n* = 120) and patients diagnosed with PLGC (*n* = 119), EGC (*n* = 143), colorectal cancer (CRC)/esophageal cancer (EC) (*n* = 42), or GC pre-/postoperatively (*n* = 46) were provided by the First Medical Center of the Chinese PLA General Hospital (Beijing, China). None of the patients were administered radiotherapy or chemotherapy treatment prior to the operation. The basic information of the patients is listed in Table 1. All plasma samples were immediately stored at −70°C. Written informed consent was obtained from all patients. The Ethics Committee of the First Medical Center of the Chinese PLA General Hospital (Beijing, China) approved the use of samples for the present study (S2017-010-02).

### 2.2. Examination of Tumour Markers

The expression levels of tumour markers CEA, alpha fetoprotein (AFP), and CA19-9 in plasma of patients were measured using the electrochemiluminescence immunoassay on the Roche Cobas 8000e602 analyser (Roche Diagnostics, Mannheim, Germany). The recommended normal reference range values for diagnostic purposes were 0–5.0 ng/ml for CEA, 0–20 ng/ml for AFP, and 0.1–37 U/ml for CA19-9. The examination was performed at the clinical laboratory of the First Medical Center of Chinese PLA General Hospital following standard procedures.

### 2.3. Plasma RNA Preparation and RT-qPCR

Plasma cfRNA was extracted from 200 *μ*L of plasma samples using the miRNeasy Serum/Plasma kit (Qiagen, Valencia, CA) according to the manufacturer's instructions. Total RNA was reverse-transcribed using ImProm-II^TM^ Reverse Transcriptase (Promega, Madison, WI) according to the manufacturer's instructions. All RNA and cDNA products were stored at −70°C. RT-qPCR was performed with the TB Green® Premix Ex Taq^TM^ (Takara, Shiga, Japan) using the Mx3000p instrument (Agilent Technologies, Santa Clara, CA) according to the manufacturer's instructions. The RNA information and primer sequences are listed in Tables S1 and S2. The expression level of each RNA was quantified using the △Ct method with the 18S rRNA gene as the endogenous control for data normalisation (Table S3), since its expression level did not significantly differ between the plasma samples of patients with GC and healthy controls [25].

### 2.4. Statistical Analyses

The statistical analyses were performed using GraphPad Prism 5.0 and IBM SPSS Statistics for Windows, version 19.0 (IBM Corp., Armonk, NY). Student's *t*-test was used for the analysis of data with normal distribution and homogeneity of variance between the two groups, whereas Mann–Whitney *U* test was used for data with skewed distribution with homogeneity of variance. All statistical tests were two-tailed, and *P* < 0.05 indicated statistical significance. The specificity, sensitivity, and area under the curve (AUC) of each RNA were determined using receiver operating characteristic (ROC) curve analysis. In addition, the expression level of RNA was analysed using logistic regression analysis to determine the diagnostic value of multiple RNA combinations. The nomogram was formulated using the R 3.5.1 software (The R Foundation for Statistical Computing, Vienna, Austria) with the *rms* statistical package.

## 3. Results

### 3.1. Positive Rates of Traditional Markers for PLGC and EGC

To test whether the traditional tumour markers could be used for the prediction of risk of GC in EGC and PLGC, we examined the levels of CEA, AFP, and CA19-9 in plasma of patients with EGC (including 129 patients) and PLGC (including 10 patients with gastricism and 101 patients with LGD or HGD) (Tables S4). We found lower positive rates for single and combined tumour markers in patients with PLGC than patients with EGC (Table 2). The positive rates of these tumour markers in both groups were lower than previously reported positive rates of tumour markers in GC patients with CEA (21.8%), AFP (5.0%), CA19-9 (24.4%), and their combinations (47.1%, including CA125) [26], which indicated that the traditional markers are unsuitable for the prediction of the risk of GC in patients with EGC and PLGC.

### 3.2. Analysis of the Expression Level of the Six RNAs in Plasma of Patients with PLGC

**Figure 1:**
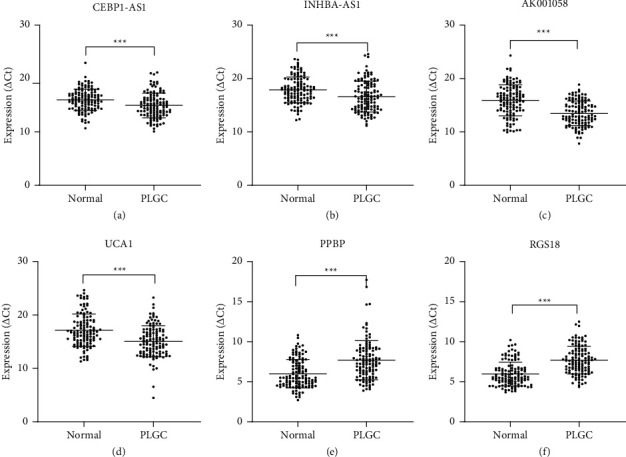
Candidate RNAs in plasma samples of patients with PLGC (*n* = 119) and healthy participants (*n* = 120). Scatter plots of plasma levels of long noncoding RNAs (a) CEBPA-AS1, (b) INHBA-AS1, (c) AK001058, and (d) UCA1, and mRNAs (e) PPBP and (f) RGS18. Expression levels of RNAs (△Ct scale *y*-axis) are normalised to that of the 18S rRNA gene. ^*∗∗∗*^*P* < 0.001. PLGC, precursor lesions of gastric cancer.

Based on our previous studies using microarray analysis [22,24], we validated the expression levels of the six RNAs in plasma samples of 119 patients with PLGC and 120 healthy participants using RT-qPCR. Of these six RNAs, CEBPA-AS1, INHBA-AS1, AK001058, and UCA1 were significantly higher and PPBP and RGS18 were significantly lower in the plasma of patients with PLGC than in that of healthy participants (*P* < 0.001) (Figure 1).

**Figure 2:**
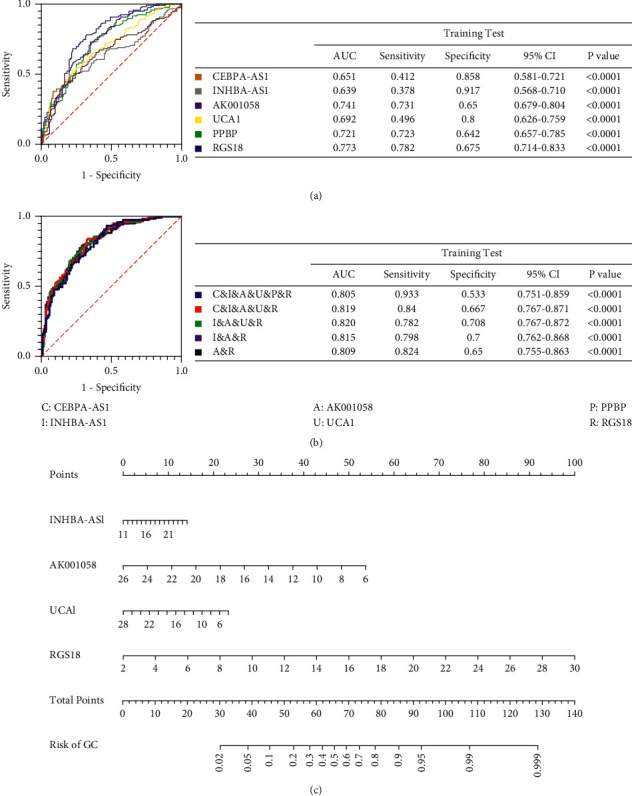
Receiver operating characteristic curve analysis of plasma RNAs to discriminate between patients with PLGC and healthy participants. (a) Receiver operating characteristic curve analysis of single plasma RNA with PPBP, RGS18, CEBPA-AS1, INHBA-AS1, AK001058, and UCA1. (b) Combined receiver operating characteristic curve analysis of abovementioned plasma RNAs. (c) A nomogram predicting the risk of GC for patients with PLGC. The value of each of variable was given a score on the point scale axis. A total score could be easily calculated by adding each single score. By projecting the total score to the lower total point scale, we were able to estimate the probability of GC. CI, confidence interval; AUC, area under the curve; (C) CEBPA-AS1; (I) INHBA-AS1; (A) AK001058; (U) UCA1; (P) PPBP; (R) RGS18; PLGC, precursor lesions of gastric cancer.

To evaluate whether plasma levels of the six candidate RNAs could be used as diagnostic biomarkers for PLGC, ROC curve analyses were performed. The plasma levels of CEBPA-AS1, INHBA-AS1, AK001058, UCA1, PPBP, and RGS18 differed between patients with PLGC and healthy participants, with AUCs of the ROC curves at 0.651, 0.639, 0.741, 0.692, 0.721, and 0.773, respectively (Figure 2(a)). Furthermore, the AUC of the combined six RNAs was 0.805 (95% confidence interval (CI): 0.751–0.859), and a six-minus-RNA signature was constructed; the six-minus-PPBP RNA signature had an AUC (AUC = 0.819; 95% CI: 0.767–0.871) higher than that for the other five RNA combinations. The combination of INHBA-AS1, AK001058, UCA1, and RGS18 (I&A&U&R) RNA signature had an AUC (AUC = 0.820; 95% CI: 0.767–0.872) higher than that of the other four RNA combinations. We also constructed three-RNA and two-RNA signatures and randomly selected from the six RNAs. Their AUCs were also lower than that of the I&A&U&R RNA signature (Figure 2(b)).

Therefore, four new RNA signatures, including INHBA-AS1, AK001058, UCA1, and RGS18, were constructed, and a nomogram was developed that incorporated the four significant risk factors for predicting GC. A total score was calculated with four RNAs, and the value of each of these variables was given a score on the point scale axis. A total score could be easily calculated by adding each single score, and by projecting the total score to the lower total point scale, we were able to estimate the probability of GC in patients with PLGC (Figure 2(c)).

### 3.3. Expression of Plasma RNAs Is Associated with Clinicopathological Features and Grade of Dysplasia in Patients with PLGC

**Figure 3:**
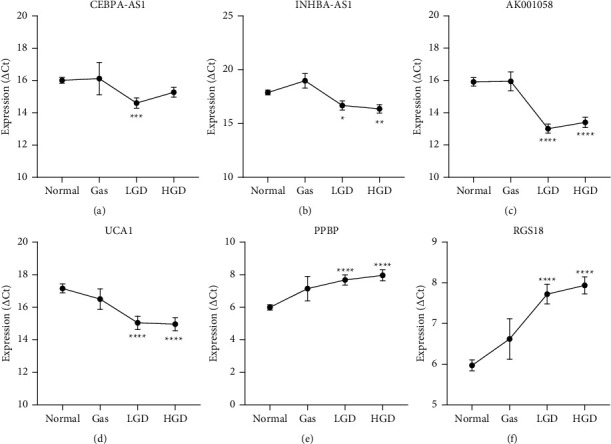
Relationship between the expression of plasma RNAs and the progression of gastric precancerous lesions. (a) CEBPA-AS1, (b) INHBA-AS1, (c) AK001058, and (d) UCA1, and mRNAs (e) PPBP and (f) RGS18. Expression levels of RNAs (△Ct scale *y*-axis) are normalised to that of the 18S rRNA gene. LGD, low-grade dysplasia; HGD, high-grade dysplasia. ^*∗*^*P* < 0.05, ^*∗∗*^*P* < 0.01, ^*∗∗∗*^*P* < 0.001,^*∗∗∗∗*^*P* < 0.0001.

The relationship between the six RNA levels in the plasma and the clinicopathological features of PLGC are shown in Table 3. The expression levels of INHBA-AS1, AK001058, UCA1, and PPBP were associated with age, and the expression level of RGS18 was associated with sex. Furthermore, we divided PLGC into gastricism, low-grade dysplasia (LGD), and high-grade dysplasia (HGD) and found that the expression of these plasma RNAs correlated with the degree of the lesion but not with gastricism (Figure 3, ^*∗*^*P* < 0.05).

### 3.4. Combination of the Six Plasma RNAs Might Predict EGC

**Figure 4:**
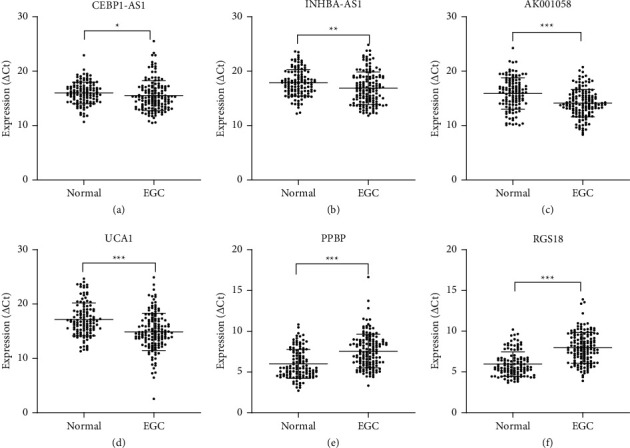
Candidate RNAs in plasma samples of patients with EGC (*n* = 143) and healthy participants (*n* = 120). Scatter plots of plasma levels of long noncoding RNAs (a) CEBPA-AS1, (b) INHBA-AS1, (c) AK001058, and (d) UCA1 and mRNAs (e) PPBP and (f) RGS18. Expression levels of RNAs (△Ct scale *y*-axis) are normalised to that of the 18S rRNA gene. ^*∗*^*P* < 0.05, ^*∗∗*^*P* < 0.01, ^*∗∗∗*^*P* < 0.001. GC, gastric cancer.

**Figure 5:**
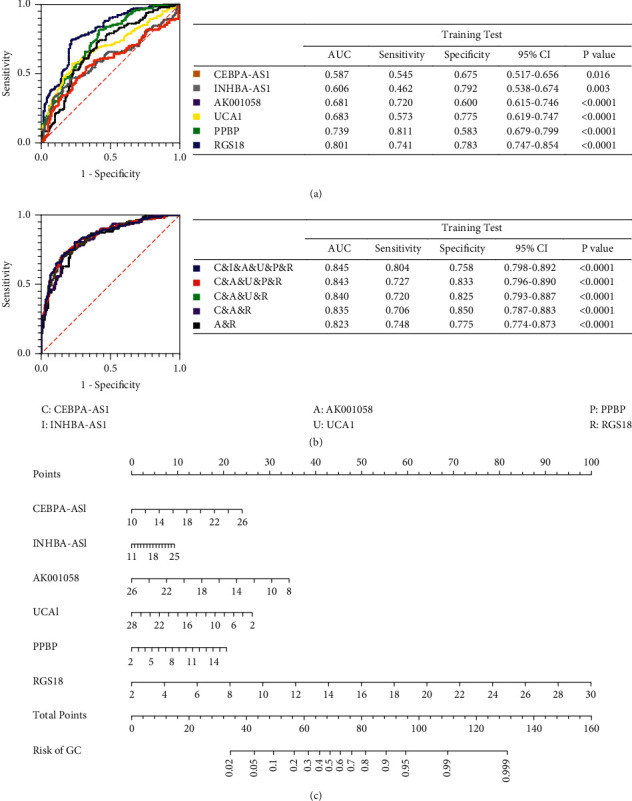
Receiver operating characteristic curve analysis of plasma RNAs discriminates patients with EGC from healthy participants. (a) Receiver operating characteristic curve analysis of single plasma RNA with PPBP, RGS18, CEBPA-AS1, INHBA-AS1, AK001058, and UCA1. (b) Combined receiver operating characteristic curve analysis of abovementioned plasma RNAs. (c) A nomogram predicting the risk of GC for patients with EGC. The value of each of variable was given a score on the point scale axis. A total score could be easily calculated by adding each single score, and by projecting the total score to the lower total point scale, we were able to estimate the probability of GC. CI, confidence interval; AUC, area under the curve; (C) CEBPA-AS1; (I) INHBA-AS1; (A) AK001058; (U) UCA1; (P) PPBP; (R) RGS18; EGC, early gastric cancer.

We validated the six RNAs using the same method in 143 patients with EGC, and the results were similar to those of patients with PLGC (Figure 4). ROC curve analyses indicated that the plasma levels of CEBPA-AS1, INHBA-AS1, AK001058, UCA1, PPBP, and RGS18 differed between patients with EGC and healthy participants, with AUCs of the ROC curves at 0.587, 0.606, 0.681, 0.683, 0.739, and 0.801, respectively (Figure 5(a)). Furthermore, the AUC of the combined six-RNA panel was 0.845 (95% CI: 0.798–0.892). We also constructed five-RNA signatures, four-RNA signatures, three-RNA signatures, and two-RNA signatures and randomly selected from the six RNAs. The resultant AUCs were lower than that for the six-RNA signature (Figure 5(b)). Therefore, the six-RNA panel, including CEBPA-AS1, INHBA-AS1, AK001058, UCA1, PPBP, and RGS18 and a nomogram that incorporated the six significant risk factors for predicting GC, were constructed. A total score was calculated with six RNAs by adding each single score. By projecting the total score to the lower total point scale, we were able to estimate the probability of GC in patients with EGC (Figure 5(c)).

### 3.5. Expression of Plasma RNAs Is Not Associated with the Clinicopathological Features and Differentiation in Patients with EGC

**Figure 6:**
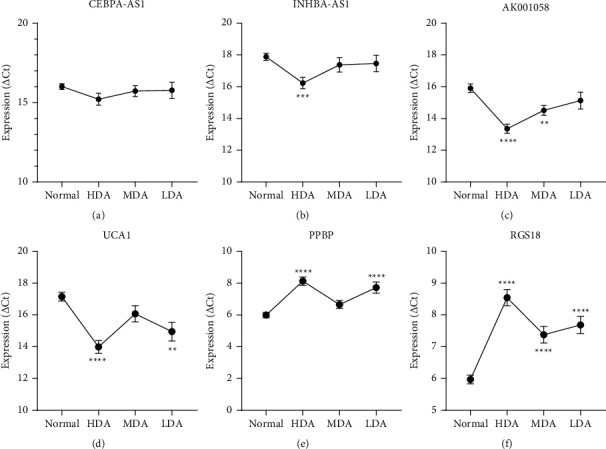
Relationship between plasma RNAs and differentiation degree of early gastric cancer. (a) CEBPA-AS1, (b) INHBA-AS1, (c) AK001058, and (d) UCA1 and mRNAs (e) PPBP and (f) RGS18. Expression levels of RNAs (△Ct scale *y*-axis) are normalised to that of the 18S rRNA gene. LDA, low-differentiation adenocarcinoma; MDA, medium-differentiation adenocarcinoma; HDA, high-differentiation adenocarcinoma. ^*∗∗*^*P* < 0.01, ^*∗∗∗*^*P* < 0.001, ^*∗∗∗∗*^*P* < 0.0001.

**Figure 7:**
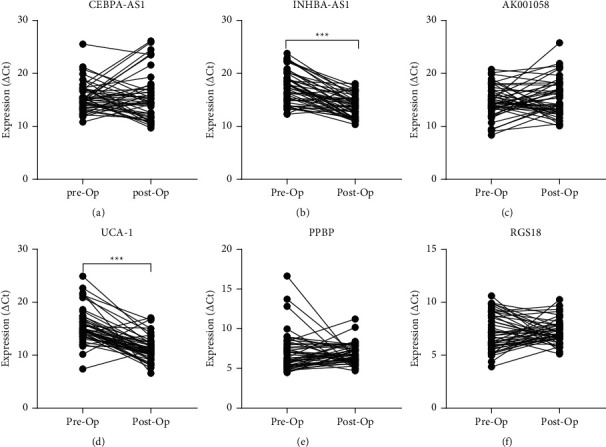
Expression of plasma RNAs pre- and postgastric cancer operation. (a) CEBPA-AS1, (b) INHBA-AS1, (c) AK001058, and (d) UCA1 and mRNAs (e) PPBP and (f) RGS18. Expression levels of RNAs (△Ct scale *y*-axis) are normalised to that of the 18S rRNA gene. Op, Operation. ^*∗∗∗*^*P* < 0.001.

The relationship between RNA levels in the plasma and the clinicopathological features of patients with EGC are shown in Table 4. There was no statistical correlation between the expression levels of the six RNAs and age, sex, and tumour size in patients with EGC. We then divided EGC into low differentiated adenocarcinoma, moderately differentiated adenocarcinoma, and highly differentiated adenocarcinoma. Although the expression of some plasma RNAs such as INHBA-AS1 and AK001058 appeared to be correlated with the differentiation degree of GC, there was no regularity in the plasma RNA expression variation in the differential stages between patients with GC and healthy controls (Figure 6). Furthermore, we analysed the expression of plasma RNAs in patients with GC pre- and postoperatively. Four RNA levels were not corrected following the operation for GC; however, the expressions of INHBA-AS1 and UCA1 were significantly lower postoperatively than preoperatively (*P* < 0.001) (Figure 7).

### 3.6. Plasma RNAs Might Be Expressed in Other Types of Gastrointestinal Cancers

**Figure 8:**
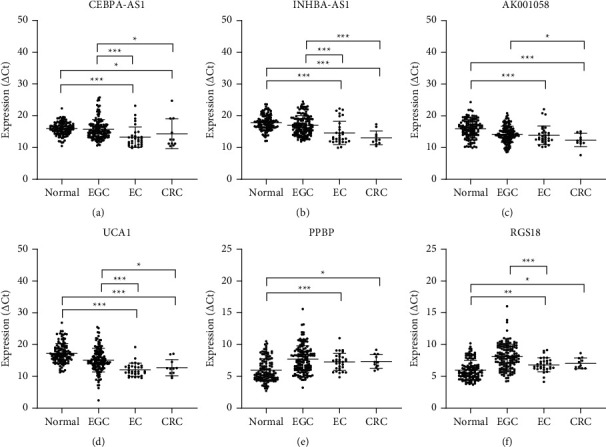
Expression of plasma RNAs in patients with CRC (*n* = 11) or EC (*n* = 31). Scatter plots of plasma levels of long noncoding RNAs (a) CEBPA-AS1, (b) INHBA-AS1, (c) AK001058, and (d) UCA1 and mRNAs (e) PPBP and (f) RGS18. Expression levels of RNAs (△Ct scale *y*-axis) are normalised to that of the 18S rRNA gene. ^*∗*^*P* < 0.05, ^*∗∗*^*P* < 0.01, ^*∗∗∗*^*P* < 0.001. CRC, colorectal cancer; EC, oesophageal cancer.

To verify whether the abovementioned six RNAs could distinguish other types of gastrointestinal cancers, plasma samples from 42 patients with other types of gastrointestinal cancers were assessed, including 11 patients with CRC and 31 patients with EC. As shown in Figure 8, the expression levels of the six RNAs showed similar pattern for patients with EC/CRC and those with EGC. However, the expression levels of CEBPA-AS1, INHBA-AS1, AK001058, and UCA1 were significantly different in the plasma of patients with EC/CRC and patients with EGC. The expression levels of the four lncRNAs were lower in patients with EGC than in healthy controls, and they were much lower in patients with EC/CRC than in healthy controls. Furthermore, although RGS18 was different between patients with EGC and EC, PPBP was not different between patients with EGC and EC/CRC, the expression levels of the two mRNAs were lower in patients with EC/CRC than in patients with EGC but were higher than that in healthy controls (Figure 8). These results are only suggestive due to the small sample size of the study, and a comparative analysis of a large sample sized study is required.

## 4. Discussion

Traditional serum markers such as CEA, CA19-9, APF, and CA125 are used widely to diagnose GC, although the positive rates of combination of these markers were 50% at most in GC [26] and much lower in EGC [5]. In recent years, cfRNA as a biomarker for early diagnosis has become a research hotspot in noninvasive diagnosis of gastric cancer, including miRNAs [27], lncRNAs [22] and mRNAs [24]. In this study, we investigated the combination of lncRNAs and mRNAs for the prediction of EGC, and we found that the combination of six RNAs (CEBPA-AS1, INHBA-AS1, AK001058, UCA1, PPBP, and RGS18) for predicting EGC and the combination of four RNAs (INHBA-AS1, AK001058, UCA1, and RGS18) for PLGC showed a better prediction than with lncRNA or mRNA alone.

As previously reported, compared with healthy participants, the expressions of lncRNA CEBPA-AS1, INHBA-AS1, AK001058, and UCA1 were significantly higher [15,22,28] and the expressions of PPBP and RGS18 were significantly lower [24] in the plasma of patients with EGC, indicating these as potential biomarkers for GC [15,22,24]. However, our results showing that the combination of these plasma lncRNAs and mRNAs rather than a single lncRNA or mRNA alone can more efficiently distinguish patients with EGC from healthy participants. Thus, the combination of different RNA molecules may predict cancer occurrence and improve the diagnostic efficiency when compared with RNA molecules used alone, as diagnostic markers. Another study reported that the combination of lncRNA H19, MEG3, and miRNA miR-675-5p improved the diagnostic efficiency compared with using each alone [27]. Although previous studies showed that most biomarkers of GC are associated with cancer differentiation [14,17,29,30], and some RNAs in this study including CEBPA-AS1, IHNBA-AS1, and AK001058 exhibited increasing trends with tumour progression, the expressions of these RNAs were not associated with GC differentiation in heathy participants which should be closed to the high differentiation in patients with GC. Our study results suggest the need to consider healthy controls when assessing the relationship between expressions of biomarkers and cancer differentiation.

In the stomach, chronic atrophic gastritis and dysplasia are two main precursor lesions preceding the development of GC [31]. However, although several studies have reported that miRNAs let-7 and miR-421 can be used for predicting PLGC [32,33], little is known about the biomarkers of PLGC. To investigate the early diagnosis of EGC, using plasma samples from patients with PLGC, the combination of four RNAs including INHBA-AS1, AK001058, UCA1, and RGS18 was analysed and found to be a candidate biomarker for the diagnosis of PLGC and indicated an increased risk of GC. Interestingly, the expressions of some of these RNAs were related to the age and sex of patients with PLGC but not those with GC, suggesting the effects of clinicopathological features on precancerous development of GC. Meanwhile, their expressions were associated with the degree of dysplasia but not with gastricism, which is in accordance with previous reports of different lncRNA expression profiles between EGC and gastritis tissues [34] and indicate an important tumorigenic transformation between gastricism and dysplasia—this requires further investigation.

We also investigated whether these plasma RNAs were differentially changed in other gastroenteric tumours including CRC and EC. We found most of them with similar patterns in CRC and EC but significantly differentially expressed compared to EGC. None of these RNAs was reported in patients with EC, while some were not reported in patients with CRC. Besides lncRNA UCA1 is upregulated and promotes CRC proliferation via the miR-143/MYO6 axis or via RNA-RNA interactions [35,36]; INHBA-AS1 is highly expressed in CRC and promotes proliferation by sponging miR-422a to increase the AKT1 axis [37]; AK001058 is significantly increased in CRC and promotes proliferation, invasion, migration, and prolongs the S stage of CRC by regulating the methylation of ADAMTS12 [38]. These results indicate that lncRNAs function in CRC and may be specific biomarkers for CRC. Together with previous reports of UCA1 upregulation in liver and pancreatic cancers [39,40] as well as CEBPA-AS1 in liver cancer [41], our results suggest that these plasma RNAs can be universal tumour markers for the diagnosis of gastrointestinal cancers. However, additional experimental studies are required to verify their expression in other gastrointestinal cancers besides GC, with a sufficient sample size to ensure consistent and repeatable results.

## 5. Conclusions

Our findings suggest that the plasma cfRNAs were differentially expressed in PLGC and EGC and that they are useful noninvasive tumour markers for the molecular diagnosis of GC. The combination of lncRNAs and mRNAs provided a higher diagnostic efficiency than single RNAs. This study has the potential to provide new insights into the application of cfRNAs as a noninvasive biomarker in large-scale screening of EGC at a precursor stage and as a general biomarker for diagnoses of gastrointestinal cancers.

## Figures and Tables

**Table 1:** Basic information of patients.

Characteristics		Data
PLGC (*n* = 119)	Age (yr)	34–86
	>60	65 (54.6%)
	<=60	54 (45.4%)
	Sex	
	Male	90 (75.6%)
	Female	29 (24.4%)
	Category	
	Gastritis	10 (8.4%)
	HGD	56 (47.1%)
	LGD	53 (44.5%)
EGC (*n* = 143)	Age (yr)	35–80
	>60	84 (58.7%)
	<=60	59 (41.3%)
	Sex	
	Male	108 (75.5%)
	Female	35 (24.5%)
	Differentiation	
	High	64 (44.7%)
	Medium	46 (32.2%)
	Low	33 (23.1%)
CRC and EC (*n* = 42)	Age (yr)	45–71
	>60	28 (66.7%)
	<=60	14 (33.3%)
	Sex	
	Male	30 (71.4%)
	Female	12 (28.6%)
	Differentiation	
	CRC high	5 (45.5%)
	CRC low	6 (54.5%)
	EC high	23 (74.2%)
	EC low	8 (25.8%)
GC with operation (*n* = 46)	Age (yr)	39–80
	>60	20 (43.5%)
	<=60	26 (56.5%)
	Sex	
	Male	33 (71.7%)
	Female	13 (28.3%)

PLGC, precursor lesions of gastric cancer; EGC, early gastric cancer; CRC, colorectal cancer; EC, esophageal cancer; GC, gastric cancer; HGD, high-grade dysplasia; LGD, low-grade dysplasia.

**Table 2:** Positive rates of single and combined tumour markers in patients with PLGC and EGC.

Tumour markers	Threshold	PLGC (*n* = 111)	EGC (*n* = 129)
CEA	>5.0 ng/ml	7 (6.3%)	17 (13.2%)
AFP	>20 ng/ml	0 (0%)	0 (0%)
CA19-9	>37 U/ml	4 (3.6%)	6 (4.7%)
CEA + AFP + CA19-9		10 (9.0%)	20 (15.5%)

PLGC, precursor lesions of gastric cancer; EGC, early gastric cancer; CEA, carcinoembryonic antigen; AFP, alpha fetoprotein.

**Table 3:** Correlation between RNA CEBPA-AS1, INHBA-AS1, AK001058, UCA1, PPBP and RGS18 panel expression levels in PLGC plasma and clinical parameters.

Clinical parameters	No. of cases	*p* value					
		CEBPA-AS1	INHBA-AS1	AK001058	UCA1	PPBP	RGS18
Age (yr)							
≤60	55	0.058	0.003^*∗∗*^	0.037^*∗*^	0.001^*∗∗*^	0.024^*∗*^	0.317
>60	64						
Sex							
Male	89	0.996	0.962	0.032	0.355	0.061	0.024^*∗*^
Female	30						
Extent of disease#							
<2 cm	22	0.141	0.088	0.156	0.256	0.185	0.317
≥2 cm	8						

^*∗*^ *P* < 0.05, ^*∗∗*^*P* < 0.01; # indicates that the analysis is carried out in an incomplete data environment. PLGC, precursor lesions of gastric cancer.

**Table 4:** Correlation between RNA CEBPA-AS1, INHBA-AS1, AK001058, UCA1, PPBP and RGS18 panel expression levels in EGC plasma and clinical parameters.

Clinical parameters	Number of cases	*p* -value					
		CEBPA-AS1	INHBA-AS1	AK001058	UCA1	PPBP	RGS18
Age (yr)							
≤60	59	0.721	0.630	0.150	0.990	0.122	0.314
>60	84						
Sex							
Male	110	0.535	0.350	0.862	0.399	0.956	0.848
Female	33						
Tumour size#							
cm	56	0.957	0.972	0.460	0.812	0.397	0.581
≥3 cm	16						

# indicates that the analysis is carried out in an incomplete data environment. EGC, early gastric cancer.

## Data Availability

The data used to support the findings of this study are available from the corresponding author upon request.

## Supplementary Materials

Supplementary Materials Table S1: information on lncRNA CEBPA-AS1, INHBA-AS1, AK001058, UCA1, and mRNA PPBP and RGS18. Table S2: sequences of primers used in the present study. Table S3: expression of plasma RNAs in patients with PLGC and EGC in the present study. Table S4: tumour markers in patients with PLGC and EGC in the present study.
