# Supplementary material for: Identification of Combinations of Plasma lncRNAs and mRNAs as Potential Biomarkers for Precursor Lesions and Early Gastric Cancer
Source: J Oncol. 2022 Feb 11;2022:1458320. doi: 10.1155/2022/1458320 (PMC8856804; doi:10.1155/2022/1458320)
Supplement: Supplementary Materials — Table S1: information on lncRNA CEBPA-AS1, INHBA-AS1, AK001058, UCA1, and mRNA PPBP and RGS18. Table S2: sequences of primers used in the present study. Table S3: expression of plasma RNAs in patients with PLGC and EGC in the present study. Table S4: tumour markers in patients with PLGC and EGC in the present study. [file 1458320.f1.zip › 1458320.f1/Table S1.docx]

**Table S1: lncRNA CEBPA-AS1, INHBA-AS1, AK001058, UCA1 and mRNA PPBP, RGS18 and related information**

| Gene Symbol | Gene type | NCBI Ref. Seq. | Location |
| --- | --- | --- | --- |
| CEBPA-AS1 (CEBPA divergent transcript) | lncRNA | NR_026887 | Chr19 |
| INHBA-AS1 (INHBA antisense RNA 1) | lncRNA | NR_027119 | Chr7 |
| lncRNA (AK001058) | lncRNA | AK001058 | Chr5 |
| UCA1 (urothelial cancer associated 1) | lncRNA | NR_015379 | Chr19 |
| PPBP (pro-platelet basic protein) | mRNA | NM_002704 | Chr4 |
| RGS18 (regulator of G-protein signaling 18) | mRNA | NM_130782 | Chr1 |

Abbreviations: lncRNA, long noncoding RNA
