# Supplementary material for: Identification of Combinations of Plasma lncRNAs and mRNAs as Potential Biomarkers for Precursor Lesions and Early Gastric Cancer
Source: J Oncol. 2022 Feb 11;2022:1458320. doi: 10.1155/2022/1458320 (PMC8856804; doi:10.1155/2022/1458320)
Supplement: Supplementary Materials — Table S1: information on lncRNA CEBPA-AS1, INHBA-AS1, AK001058, UCA1, and mRNA PPBP and RGS18. Table S2: sequences of primers used in the present study. Table S3: expression of plasma RNAs in patients with PLGC and EGC in the present study. Table S4: tumour markers in patients with PLGC and EGC in the present study. [file 1458320.f1.zip › 1458320.f1/Table S2 (1).docx]

**Table S2: Sequences of primers used in the present study.**

| Gene Symbol | Forward (5′-3′) | Reverse (5′-3′) |
| --- | --- | --- |
| CEBPA-AS1 | TGCGTCCCTCGCATTCTTTA | GACAGGAGACACTTGAGGGC |
| INHBA-AS1 | CCTACTACACACAGGGGCTC | TTCCAGAAGCTCCTCATGGG |
| AK001058 | CTGCTTTGCCATTTCCCCTT | GTTGATGCCACACAGAGGGA |
| UCA1 | AACCATCAGATCCTTGCCCA | AATATGTGGAACTGGCCCCA |
| PPBP | TGAGACAGAATGAAACAC | AGGTGATGAATCTGCTG |
| RGS18 | TGGACTAGAGGCTTTTAC | ATTTGTTGAGGTCCCTTG |
| 18S rRNA | GTAACCCGTTGAACCCCATT | CCATCCAATCGGTAGTAGCG |
