# Supplementary material for: Identification of Combinations of Plasma lncRNAs and mRNAs as Potential Biomarkers for Precursor Lesions and Early Gastric Cancer
Source: J Oncol. 2022 Feb 11;2022:1458320. doi: 10.1155/2022/1458320 (PMC8856804; doi:10.1155/2022/1458320)
Supplement: Supplementary Materials — Table S1: information on lncRNA CEBPA-AS1, INHBA-AS1, AK001058, UCA1, and mRNA PPBP and RGS18. Table S2: sequences of primers used in the present study. Table S3: expression of plasma RNAs in patients with PLGC and EGC in the present study. Table S4: tumour markers in patients with PLGC and EGC in the present study. [file 1458320.f1.zip › 1458320.f1/Table S3.docx]

**Table S3: Expression of plasma RNAs in patients with PLGC and EGC in the present study.**

| **Patient No.** | **Sex** | **Age** | **CEBPA-AS1** | **INHBA-AS1** | **AK001058** | **UCA1** | **PPBP** | **RGS18** |
| --- | --- | --- | --- | --- | --- | --- | --- | --- |
| **Healthy** |  |  |  |  |  |  |  |  |
| GIC-N-2019-001 | Male | 33 | 15.25 | 17.71 | 12.27 | 13.94 | 4.93 | 4.53 |
| GIC-N-2019-002 | Male | 46 | 13.48 | 18.97 | 12.52 | 14.06 | 4.21 | 3.86 |
| GIC-N-2019-003 | Male | 36 | 13.63 | 15.13 | 11.58 | 14.15 | 5.34 | 4.18 |
| GIC-N-2019-005 | Male | 43 | 10.49 | 12.11 | 9.93 | 11.81 | 7.31 | 6.21 |
| GIC-N-2019-006 | Male | 38 | 11.57 | 13.85 | 10.35 | 11.29 | 5.69 | 4.89 |
| GIC-N-2019-007 | Male | 37 | 12.98 | 13.76 | 11.44 | 13.17 | 10.15 | 6.49 |
| GIC-N-2019-008 | Male | 40 | 11.9 | 12.05 | 10.39 | 11.64 | 7.13 | 6.2 |
| GIC-N-2019-009 | Male | 32 | 11.64 | 16.04 | 10.11 | 12.32 | 7.66 | 9.32 |
| GIC-N-2019-010 | Male | 36 | 13.91 | 18.28 | 11.14 | 14.46 | 5.14 | 5.1 |
| GIC-N-2019-011 | Male | 40 | 11.99 | 13.27 | 10.29 | 13.81 | 4.34 | 5.67 |
| GIC-N-2019-012 | Male | 33 | 12.17 | 14.01 | 10.21 | 12.79 | 8.69 | 8.48 |
| GIC-N-2019-014 | Male | 41 | 15.95 | 13.66 | 10.31 | 15.22 | 5.59 | 4.94 |
| GIC-N-2019-016 | Male | 33 | 13.55 | 18.34 | 10.96 | 11.95 | 7.02 | 5.7 |
| GIC-N-2019-018 | Male | 42 | 15.97 | 20.23 | 12.71 | 15.28 | 4.32 | 4.4 |
| GIC-N-2019-020 | Male | 53 | 14.98 | 17.66 | 14.36 | 16.87 | 2.72 | 3.81 |
| GIC-N-2019-021 | Male | 41 | 16.41 | 21.69 | 15.55 | 15.51 | 4.41 | 4.48 |
| GIC-N-2019-022 | Male | 37 | 19.21 | 19.83 | 17.14 | 18.28 | 4.75 | 4.13 |
| GIC-N-2019-023 | Male | 34 | 15.68 | 20.34 | 14.62 | 16.75 | 6.89 | 6.4 |
| GIC-N-2019-024 | Male | 39 | 15.64 | 16.27 | 12.34 | 15.41 | 5.42 | 4.77 |
| GIC-N-2019-025 | Male | 24 | 18.95 | 22.2 | 14.09 | 16.83 | 4.44 | 5.34 |
| GIC-N-2019-026 | Male | 34 | 17.31 | 17.19 | 14.5 | 15.54 | 5.21 | 6.71 |
| GIC-N-2019-027 | Male | 38 | 17.18 | 17.89 | 14.73 | 16.49 | 5.95 | 6.6 |
| GIC-N-2019-028 | Male | 29 | 16.32 | 15.21 | 12.31 | 15.35 | 7.3 | 6.07 |
| GIC-N-2019-029 | Male | 51 | 15.39 | 16.94 | 13.44 | 15.27 | 5.07 | 5.96 |
| GIC-N-2019-031 | Male | 30 | 17.73 | 16.27 | 13.18 | 16.01 | 5.23 | 6.08 |
| GIC-N-2019-032 | Male | 35 | 17.25 | 18.3 | 14.6 | 18.17 | 4.18 | 5.46 |
| GIC-N-2019-033 | Male | 26 | 16.72 | 19.17 | 13.61 | 16.08 | 5.34 | 6.17 |
| GIC-N-2019-034 | Male | 24 | 17.75 | 18.99 | 15.74 | 19.37 | 5.13 | 28.16 |
| GIC-N-2019-035 | Male | 34 | 16.95 | 16.87 | 13.82 | 17.2 | 4.6 | 5.58 |
| GIC-N-2019-037 | Male | 31 | 14.63 | 16.46 | 13.26 | 14.13 | 3.4 | 4.46 |
| GIC-N-2019-038 | Male | 38 | 15.66 | 16.41 | 15.04 | 16.93 | 7.1 | 5.56 |
| GIC-N-2019-040 | Male | 31 | 14.89 | 15.47 | 15.96 | 15.68 | 4.99 | 6.29 |
| GIC-N-2019-041 | Male | 46 | 15.35 | 17.11 | 15.59 | 17.5 | 7.42 | 8.27 |
| GIC-N-2019-042 | Male | 45 | 15.71 | 19.91 | 15.02 | 24.23 | 7.02 | 8.39 |
| GIC-N-2019-043 | Male | 32 | 13.24 | 15.34 | 15.22 | 13.42 | 10.52 | 10.19 |
| GIC-N-2019-044 | Male | 34 | 15.3 | 14.73 | 18.53 | 16.73 | 7.84 | 8.43 |
| GIC-N-2019-045 | Male | 30 | 14.44 | 14.07 | 13.94 | 13.91 | 9.2 | 8.89 |
| GIC-N-2019-046 | Male | 49 | 14.18 | 16.44 | 16.46 | 14.72 | 7.74 | 7.87 |
| GIC-N-2019-047 | Male | 62 | 14.25 | 18.3 | 15.48 | 14.2 | 8.45 | 7.93 |
| GIC-N-2019-048 | Male | 33 | 15.57 | 19.04 | 11.38 | 16.18 | 8.48 | 8.26 |
| GIC-N-2019-049 | Male | 45 | 13.07 | 14.59 | 19.6 | 16.04 | 8.85 | 7.12 |
| GIC-N-2019-050 | Male | 61 | 13.65 | 15.44 | 21.8 | 14.99 | 9.47 | 7.9 |
| GIC-N-2019-051 | Male | 48 | 13.4 | 15.42 | 14.86 | 15.18 | 8.75 | 8.58 |
| GIC-N-2019-052 | Male | 44 | 16.01 | 16.5 | 14.01 | 19.62 | 8.68 | 7.79 |
| GIC-N-2019-053 | Male | 47 | 14.34 | 15.27 | 15.7 | 14.47 | 8.92 | 8.62 |
| GIC-N-2019-054 | Male | 21 | 15.33 | 18.13 | 18.34 | 17.86 | 8.84 | 9.34 |
| GIC-N-2019-055 | Male | 39 | 14.84 | 16.25 | 15.19 | 14.68 | 6.05 | 5.96 |
| GIC-N-2019-056 | Male | 32 | 15.09 | 16.38 | 15.09 | 15.6 | 10.03 | 8.59 |
| GIC-N-2019-058 | Male | 48 | 16.4 | 16.65 | 17.81 | 16.39 | 9.28 | 8.7 |
| GIC-N-2019-059 | Male | 37 | 14.72 | 15.75 | 19.43 | 17.94 | 4.54 | 5.59 |
| GIC-N-2019-060 | Male | 37 | 16.26 | 21.3 | 18.21 | 14.64 | 4.63 | 5.77 |
| GIC-N-2019-061 | Male | 40 | 14.47 | 16.3 | 19.88 | 16.54 | 8.21 | 8.19 |
| GIC-N-2019-062 | Male | 36 | 17.45 | 21.95 | 18.23 | 13.97 | 5.15 | 5.4 |
| GIC-N-2019-063 | Male | 27 | 16.46 | 16.98 | 16.14 | 21.79 | 4.74 | 4.53 |
| GIC-N-2019-064 | Male | 33 | 15.31 | 19.13 | 17.59 | 21.73 | 4.41 | 4.9 |
| GIC-N-2019-065 | Male | 23 | 14.15 | 17.31 | 14.04 | 11.5 | 4.75 | 5.16 |
| GIC-N-2019-066 | Male | 41 | 22.3 | 19.5 | 17.42 | 22.66 | 5.22 | 4.27 |
| GIC-N-2019-068 | Male | 47 | 15.64 | 18.46 | 14.24 | 18 | 4.79 | 5.04 |
| GIC-N-2019-069 | Male | 32 | 18.01 | 18.78 | 17.17 | 19.33 | 5.29 | 5.07 |
| GIC-N-2019-070 | Male | 40 | 16.76 | 19.47 | 18.26 | 20.86 | 4.5 | 5.31 |
| GIC-N-2019-072 | Male | 28 | 17.75 | 21.65 | 21.53 | 20.03 | 3.39 | 4.6 |
| GIC-N-2019-073 | Male | 34 | 16.78 | 21.09 | 17.73 | 23.51 | 6.14 | 4.99 |
| GIC-N-2019-074 | Male | 44 | 17.83 | 21.8 | 15.7 | 23.25 | 4.87 | 5.72 |
| GIC-N-2019-076 | Male | 44 | 15.96 | 20.77 | 16.35 | 21.48 | 4.3 | 5.07 |
| GIC-N-2019-077 | Male | 28 | 19.23 | 18.09 | 16.39 | 26.88 | 3.55 | 5.11 |
| GIC-N-2019-078 | Male | 50 | 16.36 | 20.18 | 12.6 | 20.46 | 3.44 | 4.56 |
| GIC-N-2019-079 | Male | 20 | 19.61 | 20.3 | 12.13 | 23.36 | 4.31 | 4.4 |
| GIC-N-2019-080 | Male | 32 | 18.15 | 20.63 | 15.54 | 18.42 | 4.1 | 4.35 |
| GIC-N-2019-081 | Male | 23 | 15.92 | 17.01 | 16.62 | 24.21 | 4.24 | 3.93 |
| GIC-N-2019-015 | Female | 56 | 14.33 | 16.7 | 12.58 | 14.32 | 6.54 | 4.09 |
| GIC-N-2019-017 | Female | 31 | 16.68 | 21.51 | 13.08 | 16.16 | 5.31 | 4.41 |
| GIC-N-2019-039 | Female | 37 | 14.37 | 15.55 | 20.14 | 15.56 | 3.09 | 3.69 |
| GIC-N-2019-057 | Female | 50 | 15.78 | 16 | 18.09 | 16.82 | 8.6 | 8.3 |
| GIC-N-2019-071 | Female | 45 | 17.6 | 19.81 | 19.17 | 19.58 | 4.83 | 5.56 |
| GIC-N-2019-075 | Female | 21 | 18.15 | 22.39 | 19.19 | 20.06 | 4.87 | 4.02 |
| GIC-N-2019-110 | Female | 31 | 16.57 | 16.63 | 17.26 | 16.46 | 4.99 | 6.38 |
| GIC-N-2019-112 | Female | 34 | 18.16 | 18.09 | 19.76 | 18.75 | 4.39 | 5.36 |
| GIC-N-2019-113 | Female | 28 | 16.77 | 18.27 | 16.63 | 17.31 | 7.65 | 7.01 |
| GIC-N-2019-117 | Female | 44 | 16.6 | 18.77 | 17.86 | 17.63 | 6.23 | 6.8 |
| GIC-N-2019-120 | Female | 27 | 16.6 | 18.09 | 16.95 | 17.28 | 5.48 | 5.58 |
| GIC-N-2019-125 | Female | 38 | 15.79 | 18.58 | 17.15 | 17.05 | 8.48 | 7.82 |
| GIC-N-2019-127 | Female | 56 | 15.83 | 16.48 | 16.09 | 15.16 | 8.91 | 9.65 |
| GIC-N-2019-143 | Female | 30 | 15.4 | 16.38 | 19.94 | 16.78 | 5.64 | 6.43 |
| GIC-N-2019-144 | Female | 45 | 17.5 | 18.57 | 21.85 | 16.85 | 5.48 | 5.55 |
| GIC-N-2019-146 | Female | 36 | 19.57 | 18.08 | 19.56 | 23.41 | 3.88 | 4.33 |
| GIC-N-2019-155 | Female | 34 | 18.78 | 18.19 | 20.04 | 16.16 | 6.56 | 6.62 |
| GIC-N-2019-159 | Female | 24 | 16.47 | 17.22 | 16.9 | 17.79 | 7.71 | 5.49 |
| GIC-N-2019-163 | Female | 55 | 16.2 | 16.26 | 16.78 | 17.08 | 6.7 | 6.46 |
| GIC-N-2019-177 | Female | 32 | 19.09 | 19.91 | 19.55 | 19.04 | 5.24 | 4.74 |
| GIC-N-2019-192 | Female | 36 | 18.72 | 20.63 | 18.57 | 17.61 | 7.85 | 6.74 |
| GIC-N-2019-194 | Female | 32 | 18.42 | 17.85 | 19.36 | 18.8 | 4.67 | 5.83 |
| GIC-N-2019-204 | Female | 30 | 19.17 | 21.11 | 19.27 | 19.05 | 5.01 | 5.32 |
| GIC-N-2019-216 | Female | 32 | 15.87 | 17.2 | 16.05 | 19.56 | 5.5 | 5.21 |
| GIC-N-2019-218 | Female | 21 | 16.23 | 17.94 | 19.29 | 20.38 | 4.4 | 5.01 |
| GIC-N-2019-220 | Female | 33 | 18.41 | 15.37 | 14.64 | 19.75 | 8.06 | 5.98 |
| GIC-N-2019-223 | Female | 28 | 17.9 | 17.19 | 16.64 | 17.21 | 6.21 | 5.55 |
| GIC-N-2019-232 | Female | 41 | 16.85 | 18.27 | 17.2 | 18.55 | 6.32 | 5.02 |
| GIC-N-2019-233 | Female | 34 | 14.6 | 16.73 | 16.14 | 15.72 | 6.12 | 6.27 |
| GIC-N-2019-234 | Female | 33 | 16.66 | 17.98 | 15.45 | 18.75 | 4.5 | 4.92 |
| GIC-N-2019-235 | Female | 32 | 15.14 | 17.17 | 16.09 | 18.02 | 6.16 | 5.92 |
| GIC-N-2019-236 | Female | 39 | 14.84 | 20.06 | 18.98 | 21.86 | 6.55 | 6.68 |
| GIC-N-2019-240 | Female | 43 | 14.22 | 17.59 | 15.13 | 20.98 | 6.57 | 6.67 |
| GIC-N-2019-241 | Female | 43 | 19.53 | 22.13 | 16.02 | 23.09 | 3.82 | 4.49 |
| GIC-N-2019-242 | Female | 36 | 15.71 | 17.99 | 16.79 | 23.22 | 4.72 | 5.5 |
| GIC-N-2019-244 | Female | 37 | 16.19 | 17.76 | 24.3 | 15.97 | 5.78 | 6.37 |
| GIC-N-2019-261 | Female | 46 | 15.12 | 18.58 | 16.44 | 16.36 | 9.77 | 9.03 |
| GIC-N-2019-288 | Female | 37 | 14.79 | 17.77 | 18.76 | 15.92 | 6.68 | 5.99 |
| GIC-N-2019-294 | Female | 38 | 15.15 | 18.19 | 16.64 | 14.8 | 7.01 | 6.54 |
| GIC-N-2019-307 | Female | 46 | 16.78 | 22.9 | 18.4 | 17.06 | 4.62 | 4.75 |
| GIC-N-2019-401 | Female | 22 | 17.94 | 23.61 | 20.31 | 18.6 | 5.41 | 5.45 |
| GIC-N-2019-407 | Female | 33 | 18.16 | 15.82 | 16.4 | 16.78 | 4.49 | 5.2 |
| GIC-N-2019-409 | Female | 21 | 16.84 | 15.69 | 13.98 | 16.21 | 8.16 | 7.75 |
| GIC-N-2019-410 | Female | 19 | 17.91 | 17.47 | 17.05 | 19.03 | 4.78 | 4.9 |
| GIC-N-2019-412 | Female | 27 | 17.18 | 16.42 | 16.83 | 17.02 | 5.72 | 4.07 |
| GIC-N-2019-414 | Female | 47 | 17.42 | 23.65 | 17.25 | 22.07 | 4.81 | 4.34 |
| GIC-N-2019-419 | Female | 24 | 13.39 | 18.42 | 14.42 | 14.14 | 5.82 | 6.72 |
| GIC-N-2019-420 | Female | 32 | 16.92 | 20.85 | 16.95 | 17.81 | 5.5 | 3.81 |
| GIC-N-2019-438 | Female | 30 | 14.36 | 20.6 | 18.28 | 15.28 | 5.04 | 6.29 |
| GIC-N-2019-441 | Female | 22 | 13.88 | 15.93 | 14.04 | 14.8 | 3.12 | 4.43 |
| GIC-N-2019-443 | Female | 23 | 15.79 | 16.63 | 13.89 | 20.42 | 3.64 | 4.66 |
| **Precursor Lesions of Gastric Cancer (PLGC)** | | | | | | | | |
| **Gastritis** |  |  |  |  |  |  |  |  |
| GIC-H01-2017-0167 | 68 | Female | 13.23 | 15.5 | 14.79 | 12.79 | 7.77 | 8.16 |
| GIC-H01-2018-0294 | 66 | Male | 13.94 | 16.95 | 16.94 | 16.79 | 7.28 | 8.87 |
| GIC-H01-2018-0366 | 38 | Female | 17.55 | 18.49 | 16.21 | 20.26 | 4.32 | 4.35 |
| GIC-H01-2018-0446 | 41 | Female | 21.14 | 21.12 | 18.87 | 20.32 | 4.53 | 5.47 |
| GIC-H01-2018-0466 | 60 | Female | 13 | 17.32 | 17.34 | 14.71 | 7.59 | 7.53 |
| GIC-H01-2018-0494 | 34 | Male | 16.82 | 21.06 | 16.82 | 17.37 | 5.58 | 5.24 |
| GIC-H01-2019-0029 | 58 | Female | 11.33 | 16.76 | 15.44 | 15.46 | 8.18 | 6.14 |
| GIC-H01-2019-0078 | 62 | Male | 14.7 | 18.76 | 15.98 | 15.66 | 7.38 | 5.66 |
| GIC-H01-2019-0108 | 70 | Male | 14.72 | 20.53 | 15.55 | 15.51 | 7.86 | 6.42 |
| GIC-H01-2019-0123 | 53 | Female | 15.97 | 17.54 | 14.7 | 14.45 | 5.64 | 5.62 |
| **LGD** |  |  |  |  |  |  |  |  |
| GIC-H01-2017-0023 | 73 | Female | 15.07 | 16.72 | 14.27 | 15.14 | 7.09 | 7.71 |
| GIC-H01-2017-0029 | 45 | Male | 14.11 | 15.58 | 14.94 | 18.01 | 7.89 | 8.53 |
| GIC-H01-2017-0045 | 56 | Male | 13.78 | 17.41 | 12.19 | 18.35 | 6.85 | 9.05 |
| GIC-H01-2017-0098 | 62 | Male | 13.05 | 16.17 | 13.91 | 16.39 | 7.3 | 8.48 |
| GIC-H01-2017-0100 | 43 | Male | 13.92 | 17.75 | 15 | 14.79 | 6.08 | 6.63 |
| GIC-H01-2017-0159 | 63 | Male | 12.39 | 18 | 14.04 | 16.17 | 7.61 | 7.7 |
| GIC-H01-2017-0164 | 69 | Male | 11.08 | 14.07 | 15.52 | 12.72 | 5.61 | 6.56 |
| GIC-H01-2017-0172 | 57 | Male | 13.17 | 15.52 | 8.89 | 6.55 | 10.76 | 10.15 |
| GIC-H01-2018-0006 | 60 | Female | 11.17 | 13.81 | 11.12 | 12.68 | 6.21 | 7.51 |
| GIC-H01-2018-0012 | 62 | Male | 18.49 | 14.3 | 12.88 | 16 | 6.77 | 6.7 |
| GIC-H01-2018-0047 | 63 | Male | 13.43 | 14.75 | 12.22 | 13.66 | 9.12 | 6.15 |
| GIC-H01-2018-0068 | 63 | Male | 13.63 | 16.39 | 12.65 | 17.3 | 6.41 | 6.2 |
| GIC-H01-2018-0103 | 79 | Female | 13.59 | 16.69 | 13.61 | 14.14 | 9.42 | 8 |
| GIC-H01-2018-0138 | 72 | Male | 13.75 | 14.68 | 10.81 | 14 | 11.65 | 6.65 |
| GIC-H01-2018-0157 | 65 | Male | 14.5 | 14.77 | 12.86 | 4.49 | 11.28 | 7.37 |
| GIC-H01-2018-0171 | 45 | Female | 13.12 | 15.99 | 12.39 | 16.88 | 7.21 | 7.16 |
| GIC-H01-2018-0181 | 45 | Male | 13.8 | 14.36 | 13 | 16.74 | 7.79 | 9.84 |
| GIC-H01-2018-0183 | 50 | Female | 13.92 | 13.08 | 13.77 | 15.51 | 9.34 | 9.17 |
| GIC-H01-2018-0189 | 56 | Male | 12.46 | 13.57 | 11.69 | 17.43 | 8.44 | 8.45 |
| GIC-H01-2018-0229 | 53 | Male | 19.04 | 15.7 | 12.7 | 12.05 | 8.48 | 8.84 |
| GIC-H01-2018-0231 | 52 | Male | 14.7 | 15.3 | 13.2 | 13.39 | 7.3 | 8.3 |
| GIC-H01-2018-0232 | 52 | Female | 13.51 | 14.27 | 12.86 | 13.92 | 6.31 | 6.42 |
| GIC-H01-2018-0251 | 47 | Female | 13.98 | 19.84 | 13.76 | 14.97 | 9.14 | 8.85 |
| GIC-H01-2018-0256 | 67 | Male | 12.43 | 13.61 | 10.8 | 16.65 | 8.16 | 7.43 |
| GIC-H01-2018-0261 | 55 | Female | 13 | 16.15 | 13.97 | 15.2 | 8.87 | 6.8 |
| GIC-H01-2018-0270 | 70 | Female | 15.31 | 12 | 9.7 | 9.8 | 7.73 | 6.7 |
| GIC-H01-2018-0301 | 57 | Male | 16.19 | 18.92 | 14.88 | 16.53 | 6.48 | 7.15 |
| GIC-H01-2018-0337 | 66 | Male | 11.61 | 12.47 | 10.03 | 12.41 | 5.09 | 5.99 |
| GIC-H01-2018-0365 | 60 | Male | 18.87 | 20.8 | 16.53 | 18.9 | 4.82 | 7.79 |
| GIC-H01-2018-0374 | 48 | Female | 14.4 | 19.48 | 17.1 | 19 | 5.23 | 4.58 |
| GIC-H01-2018-0382 | 64 | Male | 15.66 | 14.16 | 11.19 | 15.1 | 6.91 | 7.23 |
| GIC-H01-2018-0395 | 71 | Male | 17.88 | 21.1 | 16.07 | 16.92 | 4.97 | 7.38 |
| GIC-H01-2018-0413 | 67 | Male | 13.47 | 13.75 | 11.41 | 12.44 | 5.18 | 5.61 |
| GIC-H01-2018-0448 | 61 | Male | 11.52 | 19.55 | 10.76 | 11.95 | 4.07 | 5.26 |
| GIC-H01-2018-0493 | 47 | Male | 15.14 | 20.41 | 12.93 | 15.77 | 8.21 | 6.02 |
| GIC-H01-2018-0495 | 63 | Male | 20.78 | 16.66 | 17.01 | 17.23 | 7.8 | 5.25 |
| GIC-H01-2018-0507 | 66 | Male | 10.1 | 14.22 | 10.56 | 11.71 | 10.93 | 5.97 |
| GIC-H01-2018-0509 | 60 | Male | 14.43 | 16.73 | 12.6 | 13.67 | 4.1 | 4.91 |
| GIC-H01-2019-0007 | 59 | Female | 15.83 | 19.96 | 14.82 | 17.01 | 5.21 | 7.11 |
| GIC-H01-2019-0017 | 52 | Male | 14.34 | 21.51 | 14.76 | 18.37 | 4.58 | 6.42 |
| GIC-H01-2019-0022 | 57 | Female | 15.51 | 14.99 | 10.74 | 14.52 | 7.24 | 6.61 |
| GIC-H01-2019-0087 | 46 | Female | 17.34 | 18.3 | 14.65 | 17.29 | 6.54 | 7.94 |
| GIC-H01-2019-0090 | 66 | Male | 10.62 | 11.16 | 7.81 | 10.69 | 5.05 | 4.82 |
| GIC-H01-2019-0106 | 56 | Female | 18.16 | 20.79 | 15.8 | 18.4 | 4.65 | 6.97 |
| GIC-H01-2019-0109 | 60 | Male | 19.31 | 24.56 | 16 | 18.53 | 4.48 | 7.76 |
| GIC-H02-@025A | 76 | Male | 13.14 | 15.69 | 11.9 | 16.04 | 7.97 | 9.48 |
| GIC-H02-019 | 58 | Male | 16.1 | 17.63 | 13.84 | 16 | 10.56 | 12.5 |
| GIC-H02-030 | 76 | Male | 14.9 | 15.03 | 12 | 15.26 | 9.9 | 10.02 |
| GIC-H02-043 | 74 | Male | 16.13 | 14.71 | 13.25 | 15.77 | 7.38 | 9.54 |
| GIC-H02-044 | 70 | Male | 13.73 | 16.36 | 12.53 | 14.21 | 8.3 | 9.5 |
| GIC-H02-073 | 68 | Male | 13.27 | 15.74 | 12.83 | 14.43 | 12.13 | 11.97 |
| GIC-H02-080 | 42 | Male | 14.69 | 24.31 | 12.28 | 21.99 | 14.64 | 10.21 |
| GIC-H02-128 | 49 | Male | 14.92 | 16.15 | 12.42 | 14.16 | 10.39 | 12.15 |
| **HGD** |  |  |  |  |  |  |  |  |
| GIC-H01-2017-0019 | 45 | Male | 15.93 | 19.68 | 10.94 | 14.69 | 14.71 | 7.62 |
| GIC-H01-2017-0049 | 61 | Male | 19.51 | 18.23 | 16.22 | 19.65 | 4.94 | 6.91 |
| GIC-H01-2017-0129 | 84 | Male | 16.51 | 18.53 | 15.12 | 15.38 | 5.99 | 6.48 |
| GIC-H01-2018-0059 | 45 | Male | 17.34 | 17.43 | 15.33 | 17.41 | 5.4 | 7.89 |
| GIC-H01-2018-0069 | 59 | Male | 15.05 | 16.77 | 12.25 | 15.49 | 3.89 | 4.85 |
| GIC-H01-2018-0081 | 51 | Male | 18.56 | 19.59 | 17.88 | 19.66 | 6.32 | 8 |
| GIC-H01-2018-0090 | 51 | Male | 16.34 | 22.28 | 14.57 | 17.77 | 5.47 | 6.4 |
| GIC-H01-2018-0102 | 53 | Male | 15.66 | 16.45 | 15.94 | 15.61 | 7.47 | 5.81 |
| GIC-H01-2018-0132 | 54 | Male | 17.46 | 19.03 | 16.16 | 18.49 | 4.64 | 6.27 |
| GIC-H01-2018-0197 | 74 | Male | 17.24 | 16.15 | 13.93 | 21.37 | 6.98 | 7.23 |
| GIC-H01-2018-0203 | 50 | Male | 15.59 | 16.27 | 11.73 | 15.26 | 17.72 | 7.83 |
| GIC-H01-2018-0338 | 48 | Male | 15.41 | 17.05 | 14.24 | 16.65 | 6.34 | 7.63 |
| GIC-H01-2018-0356 | 64 | Male | 15.58 | 24.04 | 12.92 | 14.79 | 5.96 | 4.73 |
| GIC-H01-2019-0075 | 55 | Male | 17.78 | 19.41 | 16.39 | 19.57 | 7.84 | 7.64 |
| GIC-H01-2019-0079 | 61 | Male | 17.72 | 19.16 | 16.84 | 18.11 | 9.21 | 6.15 |
| GIC-H01-2019-0095 | 62 | Male | 11.85 | 12.48 | 8.9 | 12.2 | 16.84 | 10.72 |
| GIC-H01-2019-0128 | 61 | Male | 16.78 | 17.35 | 14.74 | 17.31 | 6.71 | 8 |
| GIC-H01-2019-0133 | 63 | Male | 17.58 | 17.46 | 14.8 | 16.3 | 5.5 | 7.2 |
| GIC-H02-@028A | 69 | Male | 15.23 | 14.56 | 10.89 | 12.29 | 9.21 | 10.11 |
| GIC-H02-006 | 69 | Male | 13.63 | 12.84 | 12.18 | 12.4 | 9.19 | 10.11 |
| GIC-H02-060 | 61 | Male | 13.88 | 12.68 | 10.38 | 12.31 | 7.54 | 8.87 |
| GIC-H02-086 | 48 | Male | 14.34 | 14.94 | 10.82 | 13.16 | 6.86 | 7.95 |
| GIC-H02-091 | 62 | Male | 14.9 | 13.06 | 12.16 | 12.84 | 9.57 | 10.54 |
| GIC-H02-111 | 63 | Male | 14.17 | 13.19 | 11.91 | 13.14 | 8.77 | 10.02 |
| GIC-H02-119 | 63 | Male | 12.91 | 18.71 | 11.16 | 13.14 | 10.05 | 9.69 |
| GIC-H02-136 | 56 | Male | 15.35 | 13.69 | 11.66 | 13.75 | 6.63 | 8.47 |
| GIC-H02-137 | 55 | Male | 14.56 | 18.83 | 12.24 | 13.92 | 6.83 | 7.66 |
| GIC-H08-2019-0159 | 47 | Male | 14.08 | 14.62 | 12.91 | 14.49 | 12.32 | 8.14 |
| GIC-H08-2019-0349 | 46 | Male | 13.88 | 14.3 | 14.01 | 12.08 | 9.6 | 8.84 |
| GIC-H08-2019-0415 | 72 | Male | 16.34 | 16.95 | 16.37 | 18.89 | 8.99 | 9.12 |
| GIC-H01-2017-0136 | 50 | Male | 19.35 | 13.3 | 13.88 | 12.33 | 6.44 | 6.89 |
| GIC-H01-2018-0052 | 74 | Male | 12.53 | 17.58 | 14.48 | 12.94 | 9.05 | 6.75 |
| GIC-H01-2018-0032 | 68 | Male | 17.46 | 18.54 | 17.56 | 12.53 | 5.93 | 5.96 |
| GIC-H01-2018-0309 | 78 | Male | 16.41 | 19.85 | 16.45 | 10.43 | 9.82 | 9.58 |
| GIC-H01-2018-0436 | 67 | Male | 12.03 | 13.61 | 11.71 | 14.46 | 8.36 | 7.68 |
| GIC-H01-2018-0450 | 82 | Male | 11.41 | 12.67 | 11.33 | 13.31 | 8.71 | 6.91 |
| GIC-H01-2018-0513 | 67 | Male | 15.75 | 19.98 | 16.36 | 19.13 | 6.12 | 6.49 |
| GIC-H01-2019-0138 | 63 | Male | 16.93 | 19.72 | 12.74 | 13.87 | 6.36 | 7.82 |
| GIC-H02-057 | 86 | Male | 12.73 | 14.92 | 10.88 | 13.14 | 9 | 10.51 |
| GIC-H02-095 | 71 | Male | 14.14 | 19.16 | 10.46 | 12.32 | 9.63 | 10.31 |
| GIC-H02-100 | 49 | Male | 12.15 | 14.25 | 10.17 | 13.14 | 8.88 | 10.53 |
| GIC-H02-120 | 69 | Male | 11.64 | 14.03 | 11.5 | 12.92 | 7.62 | 9.1 |
| GIC-H02-131 | 63 | Male | 11.38 | 13.44 | 9.79 | 12.13 | 9.29 | 10.51 |
| GIC-H01-2017-0017 | 67 | Male | 20.99 | 14.55 | 12.88 | 12.97 | 11.79 | 10.79 |
| GIC-H01-2017-0195 | 66 | Male | 14.86 | 13.55 | 11.65 | 14.47 | 7.94 | 8.22 |
| GIC-H01-2018-0045 | 72 | Male | 15.29 | 12.63 | 12.31 | 12.23 | 7.16 | 8.58 |
| GIC-H01-2018-0070 | 79 | Female | 18.93 | 21 | 17.4 | 20.02 | 5.48 | 7.31 |
| GIC-H01-2018-0346 | 51 | Female | 15.31 | 19.55 | 15.17 | 16.23 | 5.71 | 6.76 |
| GIC-H01-2018-0474 | 54 | Female | 15.26 | 16.65 | 14.72 | 17.09 | 7.16 | 6.9 |
| GIC-H01-2018-0497 | 66 | Female | 17.18 | 20.19 | 17.07 | 23.26 | 7.19 | 5.97 |
| GIC-H02-@021A | 67 | Female | 13.19 | 13.19 | 12.49 | 12.17 | 5.44 | 5.8 |
| GIC-H02-@027A | 72 | Female | 12.16 | 14.31 | 9.87 | 12.6 | 7.88 | 8.57 |
| GIC-H08-2018-0157 | 56 | Female | 16 | 15.32 | 15.57 | 14.5 | 5.16 | 5.52 |
| GIC-H08-2019-0399 | 67 | Female | 12.9 | 11.46 | 11.01 | 10.02 | 8.91 | 9.67 |
| GIC-H02-@018A | 52 | Female | 16.13 | 14.68 | 12.34 | 15.73 | 6.52 | 8.54 |
| GIC-H01-2018-0122 | 75 | Female | 15.37 | 14.05 | 16.04 | 13.71 | 9.2 | 7.35 |
| **Early Gastric Cancer (EGC)** | | | | | | | | |
| **LDA** |  |  |  |  |  |  |  |  |
| GIC-H01-2018-0159 | 62 | Female | 14.55 | 22.79 | 19.13 | 21.89 | 5.37 | 6.12 |
| GIC-H01-2018-0165 | 75 | Female | 15.15 | 18.93 | 13.81 | 15.31 | 15.6 | 16.03 |
| GIC-H08-2019-0177 | 58 | Female | 14.73 | 12.59 | 12.94 | 14.19 | 7.62 | 7.79 |
| GIC-H08-2019-0187 | 59 | Male | 12.1 | 17.69 | 13.9 | 11.99 | 8.98 | 9.05 |
| GIC-H08-2019-0203 | 51 | Male | 14.85 | 17.46 | 16.84 | 21.43 | 7.61 | 8.26 |
| GIC-H08-2019-0207 | 62 | Male | 17.35 | 16.56 | 20.81 | 10.22 | 7.09 | 7.89 |
| GIC-H08-2019-0231 | 56 | Female | 18.87 | 20.7 | 20.15 | 20.87 | 5.64 | 5.19 |
| GIC-H08-2019-0273 | 74 | Male | 13.35 | 19.19 | 15.12 | 15.87 | 8.98 | 8.73 |
| GIC-H08-2019-0291 | 49 | Female | 14.33 | 18.99 | 15.78 | 15.03 | 8.44 | 8.23 |
| GIC-H08-2019-0341 | 75 | Male | 14.81 | 18.47 | 17.89 | 15.75 | 9.24 | 10.85 |
| **MDA** |  |  |  |  |  |  |  |  |
| GIC-H01-2017-0082 | 67 | Male | 13.51 | 16.2 | 13.06 | 8.68 | 7.25 | 7.29 |
| GIC-H01-2017-0096 | 64 | Male | 18.12 | 16.85 | 14.5 | 10.59 | 7.52 | 7.93 |
| GIC-H01-2017-0117 | 66 | Male | 13.26 | 12.84 | 15.45 | 12.26 | 6.44 | 7.75 |
| GIC-H01-2017-0197 | 50 | Male | 15.21 | 13.35 | 16.17 | 8.19 | 7.76 | 8.2 |
| GIC-H01-2018-0034 | 45 | Male | 20.39 | 16.66 | 13.97 | 9.08 | 8.54 | 7.25 |
| GIC-H01-2018-0082 | 63 | Male | 17.26 | 18.99 | 18.49 | 15.09 | 5 | 6.07 |
| GIC-H01-2018-0083 | 35 | Male | 13.85 | 22.3 | 15.41 | 20.16 | 6.49 | 7.48 |
| GIC-H01-2018-0131 | 72 | Male | 15.2 | 18.37 | 14.35 | 14.98 | 5.25 | 6.17 |
| GIC-H01-2018-0169 | 63 | Female | 23.13 | 22.97 | 17.3 | 17.89 | 6.83 | 7.92 |
| GIC-H01-2018-0188 | 62 | Male | 11.04 | 12.43 | 8.57 | 12.22 | 13.18 | 10.17 |
| GIC-H01-2018-0207 | 64 | Male | 17.26 | 18.15 | 15.23 | 14.14 | 6.21 | 7.37 |
| GIC-H01-2018-0239 | 58 | Male | 13.55 | 13.8 | 13.87 | 14.62 | 5.54 | 6.84 |
| GIC-H01-2018-0241 | 63 | Female | 14.61 | 14.37 | 9.11 | 13.71 | 4.45 | 6.1 |
| GIC-H01-2018-0250 | 60 | Female | 13.68 | 13.65 | 10.8 | 12.57 | 5.88 | 6.91 |
| GIC-H01-2018-0313 | 59 | Male | 12.26 | 22.17 | 15.54 | 14.41 | 5.05 | 6.6 |
| GIC-H01-2018-0343 | 48 | Male | 16.85 | 15.96 | 18.24 | 21.43 | 4.89 | 7.19 |
| GIC-H01-2018-0353 | 71 | Female | 16.59 | 20 | 13.22 | 16.06 | 5.18 | 5.64 |
| GIC-H01-2018-0411 | 39 | Male | 21.36 | 23.58 | 19.81 | 25.18 | 4.49 | 5.86 |
| GIC-H01-2018-0473 | 54 | Male | 18 | 20.02 | 14.98 | 17.98 | 6.42 | 7.11 |
| GIC-H01-2018-0514 | 60 | Male | 15.06 | 15.99 | 11.8 | 14.26 | 6.6 | 5.15 |
| GIC-H01-2019-0012 | 57 | Male | 24.93 | 22.98 | 13.8 | 15.91 | 7.19 | 9.49 |
| GIC-H01-2019-0018 | 64 | Male | 15.36 | 15.67 | 10 | 15.01 | 5.85 | 6.53 |
| GIC-H01-2019-0026 | 69 | Male | 16.03 | 22.78 | 14.37 | 19.23 | 8.74 | 8.7 |
| GIC-H01-2019-0041 | 72 | Male | 16.64 | 15.39 | 13.35 | 14.17 | 8.29 | 8.46 |
| GIC-H01-2019-0054 | 72 | Male | 13.82 | 13.13 | 11.83 | 13.41 | 5.96 | 4.29 |
| GIC-H01-2019-0061 | 77 | Male | 18.11 | 18.55 | 15.36 | 8.87 | 6.63 | 5.35 |
| GIC-H01-2019-0064 | 74 | Female | 16.51 | 19.11 | 16.3 | 21.42 | 7.33 | 7.77 |
| GIC-H01-2019-0085 | 70 | Female | 16.06 | 17.41 | 13.17 | 17.89 | 8.12 | 5.91 |
| GIC-H01-2019-0115 | 58 | Male | 18.39 | 22 | 13.13 | 15.93 | 6.31 | 6.25 |
| GIC-H01-2019-0118 | 69 | Male | 17 | 19.06 | 15.51 | 17.49 | 5.11 | 7.38 |
| GIC-H01-2019-0158 | 69 | Female | 16.78 | 19.87 | 13.93 | 17.62 | 5.14 | 7.11 |
| GIC-H02-008 | 60 | Male | 17.28 | 15.55 | 13.58 | 14.81 | 8.08 | 9.81 |
| GIC-H02-021 | 53 | Male | 15.14 | 14.74 | 11.67 | 14.07 | 8.41 | 9.69 |
| GIC-H02-085 | 79 | Female | 14.16 | 15.08 | 12.83 | 13.98 | 6.81 | 8.1 |
| GIC-H02-087 | 52 | Male | 12.26 | 14.62 | 11 | 13.95 | 8.53 | 10.43 |
| GIC-H02-104 | 64 | Male | 13.27 | 13.82 | 11.24 | 12.96 | 11.9 | 10.91 |
| GIC-H02-116 | 79 | Female | 12.62 | 15.17 | 13.47 | 15.14 | 8.25 | 8.06 |
| GIC-H02-121 | 61 | Female | 12.85 | 16.9 | 14.84 | 15.81 | 10.61 | 9.87 |
| GIC-H08-2019-0165 | 44 | Female | 13.02 | 15.56 | 15.18 | 15.02 | 7.32 | 7.17 |
| GIC-H08-2019-0185 | 53 | Female | 15.55 | 13.64 | 16.65 | 13.44 | 8.48 | 9.17 |
| GIC-H08-2019-0255 | 62 | Male | 17.35 | 16.22 | 17.18 | 16.13 | 6.66 | 5.26 |
| GIC-H08-2019-0257 | 67 | Female | 17.19 | 19 | 12.19 | 22.7 | 6.36 | 6.15 |
| GIC-H08-2019-0267 | 80 | Male | 15.31 | 17.15 | 14.8 | 16.88 | 5.82 | 5.52 |
| GIC-H08-2019-0275 | 61 | Male | 15.52 | 15.66 | 15.03 | 12.74 | 6.05 | 5.86 |
| GIC-H08-2019-0299 | 57 | Male | 12.99 | 16.24 | 18.12 | 17.33 | 7.24 | 7.08 |
| GIC-H08-2019-0303 | 67 | Male | 13.2 | 16.44 | 15.79 | 14.74 | 8.19 | 6.89 |
| GIC-H08-2019-0325 | 62 | Female | 18.26 | 18.99 | 15.98 | 18.07 | 8.88 | 9.81 |
| GIC-H08-2019-0351 | 51 | Male | 11.95 | 13.16 | 13.14 | 12.93 | 8.77 | 9.61 |
| GIC-H08-2019-0357 | 48 | Male | 17.17 | 20.83 | 18.46 | 18.36 | 8.44 | 9.84 |
| GIC-H08-2019-0359 | 57 | Female | 16.86 | 20.08 | 16.4 | 23.91 | 9.3 | 10.28 |
| GIC-H08-2019-0363 | 62 | Male | 17.11 | 13.68 | 14.44 | 14.53 | 9.88 | 10.55 |
| GIC-H08-2019-0373 | 50 | Male | 14.64 | 16.26 | 17.32 | 15.38 | 6.68 | 5.82 |
| GIC-H08-2019-0375 | 50 | Male | 15.09 | 17.46 | 16.45 | 16.94 | 8.33 | 8.68 |
| GIC-H08-2019-0379 | 51 | Male | 15.1 | 14.92 | 11.35 | 16.63 | 6.69 | 8.39 |
| GIC-H08-2019-0401 | 52 | Male | 14.44 | 17.54 | 18.17 | 14.42 | 7.49 | 8.46 |
| **HDA** |  |  |  |  |  |  |  |  |
| GIC-H01-2017-0014 | 61 | Male | 17.42 | 15.09 | 9.19 | 10.31 | 10.93 | 7.12 |
| GIC-H01-2017-0036 | 74 | Male | 19.25 | 16.19 | 11.42 | 14.43 | 11.94 | 9.32 |
| GIC-H01-2017-0051 | 67 | Male | 21.21 | 17.14 | 13.46 | 14.17 | 6.25 | 7.62 |
| GIC-H01-2017-0081 | 51 | Male | 14.58 | 15.66 | 13.89 | 6.31 | 6.9 | 7.74 |
| GIC-H01-2017-0130 | 50 | Female | 21.96 | 13.29 | 13.35 | 7.17 | 8.29 | 7.94 |
| GIC-H01-2017-0211 | 51 | Female | 13.65 | 14.27 | 13.88 | 2.45 | 8.14 | 8.25 |
| GIC-H01-2018-0001 | 65 | Male | 16.16 | 22.62 | 16.68 | 23.79 | 4.45 | 5.84 |
| GIC-H01-2018-0024 | 48 | Male | 15.43 | 14.9 | 13.49 | 14.73 | 8.17 | 10.07 |
| GIC-H01-2018-0029 | 72 | Male | 15.89 | 18.28 | 14.3 | 17.73 | 6.36 | 7.17 |
| GIC-H01-2018-0196 | 43 | Female | 15.15 | 15.44 | 13.12 | 14.37 | 5.12 | 5.57 |
| GIC-H01-2018-0230 | 48 | Male | 13.38 | 14.05 | 13.91 | 22.02 | 5.43 | 6.3 |
| GIC-H01-2018-0233 | 58 | Male | 21.85 | 24.46 | 18.2 | 20.66 | 5.02 | 5.45 |
| GIC-H01-2018-0249 | 57 | Male | 14.42 | 15.75 | 14.85 | 14.31 | 6.05 | 6.78 |
| GIC-H01-2018-0295 | 57 | Female | 18.91 | 19.39 | 15.03 | 18.59 | 10.21 | 7.27 |
| GIC-H01-2018-0308 | 44 | Male | 12.48 | 15.17 | 14.11 | 14.38 | 4.97 | 6.65 |
| GIC-H01-2018-0324 | 70 | Male | 16.75 | 22.02 | 17.79 | 25.54 | 4.66 | 5.29 |
| GIC-H01-2018-0373 | 73 | Male | 25.77 | 17.57 | 19.24 | 18.11 | 5.07 | 5.63 |
| GIC-H01-2018-0377 | 78 | Male | 25.5 | 19.37 | 13.95 | 15.47 | 3.21 | 4.75 |
| GIC-H01-2018-0406 | 78 | Male | 21.18 | 16.6 | 13.92 | 13.46 | 5.16 | 6.89 |
| GIC-H01-2018-0414 | 74 | Male | 12.18 | 15.31 | 11.8 | 13.82 | 7.41 | 8.98 |
| GIC-H01-2018-0420 | 59 | Female | 24.8 | 22.84 | 18.06 | 7.24 | 4.88 | 5.67 |
| GIC-H01-2018-0428 | 60 | Male | 15.7 | 14.27 | 15.96 | 9.65 | 6.1 | 6.69 |
| GIC-H01-2018-0445 | 70 | Male | 22.9 | 19.38 | 12.33 | 9.96 | 9 | 8.54 |
| GIC-H01-2018-0477 | 64 | Male | 16.61 | 22.36 | 15.31 | 17.5 | 13.12 | 8.12 |
| GIC-H01-2019-0013 | 54 | Male | 18.56 | 23.79 | 15.27 | 19.66 | 8.31 | 7.79 |
| GIC-H01-2019-0016 | 55 | Male | 15.48 | 20.64 | 13.96 | 17.14 | 5.19 | 4.22 |
| GIC-H01-2019-0020 | 61 | Male | 19.88 | 20.94 | 9.45 | 15.35 | 6.14 | 9.93 |
| GIC-H01-2019-0028 | 70 | Male | 20.52 | 14.79 | 11.7 | 14.12 | 6.79 | 6.42 |
| GIC-H01-2019-0040 | 70 | Male | 15.28 | 17.05 | 14.58 | 15.9 | 5.58 | 4.72 |
| GIC-H01-2019-0044 | 48 | Male | 14.84 | 19.69 | 11.54 | 12.27 | 8.32 | 8.29 |
| GIC-H01-2019-0055 | 55 | Male | 14.36 | 17.6 | 13.96 | 16.17 | 5.94 | 5.08 |
| GIC-H01-2019-0056 | 55 | Male | 15.17 | 17.23 | 14.15 | 17.2 | 6.16 | 5.83 |
| GIC-H01-2019-0069 | 67 | Male | 15.64 | 16.47 | 13.07 | 15.64 | 4.77 | 6.33 |
| GIC-H01-2019-0072 | 52 | Male | 14.17 | 16.33 | 12.81 | 16.41 | 6.01 | 5.54 |
| GIC-H01-2019-0122 | 62 | Male | 14.79 | 21.33 | 13.36 | 16.28 | 6.02 | 7.74 |
| GIC-H01-2019-0124 | 74 | Female | 16.47 | 17.02 | 14.59 | 17.39 | 7.19 | 7.52 |
| GIC-H01-2019-0136 | 70 | Female | 16.86 | 20.62 | 14.08 | 19.35 | 10.55 | 10.57 |
| GIC-H01-2019-0139 | 64 | Male | 18.54 | 22.03 | 16.8 | 19.5 | 11.62 | 9.54 |
| GIC-H01-2019-0162 | 54 | Male | 17.35 | 19.83 | 15.88 | 19.31 | 4.84 | 7.06 |
| GIC-H02-@001A | 70 | Male | 14.58 | 18.89 | 13.87 | 20.21 | 9.5 | 10.54 |
| GIC-H02-@009A | 64 | Male | 16.59 | 13.98 | 13.59 | 13.87 | 9.65 | 8.05 |
| GIC-H02-@012A | 62 | Male | 11.68 | 13.25 | 14.91 | 12.17 | 8.12 | 9.7 |
| GIC-H02-@014A | 64 | Male | 13.15 | 13.92 | 13.85 | 16.1 | 9.01 | 8.74 |
| GIC-H02-@017A | 72 | Male | 13.41 | 18.37 | 14.31 | 14.64 | 9.02 | 8.91 |
| GIC-H02-@022A | 75 | Male | 12.66 | 17.73 | 17.79 | 8.93 | 10.42 | 9.11 |
| GIC-H02-@024A | 62 | Male | 14.74 | 14.43 | 11.97 | 12.48 | 9.41 | 8.64 |
| GIC-H02-@026A | 64 | Male | 14.13 | 12.81 | 12.47 | 12.48 | 6.96 | 7.62 |
| GIC-H02-007 | 71 | Male | 13.44 | 14.32 | 10.67 | 14.13 | 8.56 | 9.51 |
| GIC-H02-020 | 50 | Male | 13.21 | 15.21 | 11.03 | 13.07 | 10.9 | 11 |
| GIC-H02-022 | 67 | Male | 16.85 | 14.95 | 14.39 | 15.07 | 7.68 | 10.21 |
| GIC-H02-031 | 55 | Male | 13.66 | 18.92 | 11.84 | 13.52 | 8.43 | 10.08 |
| GIC-H02-040 | 58 | Male | 14.51 | 16.17 | 12.67 | 14.07 | 10.09 | 13.87 |
| GIC-H02-041 | 54 | Male | 14.86 | 15.84 | 12.75 | 14.07 | 6.33 | 9.3 |
| GIC-H02-078 | 61 | Male | 14.46 | 14.16 | 9.56 | 11.39 | 10.82 | 13.5 |
| GIC-H02-088 | 73 | Female | 14.03 | 17.58 | 12.87 | 13.96 | 6.25 | 7.49 |
| GIC-H02-090 | 58 | Male | 13.74 | 16.88 | 10.15 | 12.93 | 8.49 | 9.71 |
| GIC-H02-093 | 61 | Male | 18.85 | 13.83 | 8.67 | 11.06 | 9.77 | 12.2 |
| GIC-H02-094 | 51 | Male | 11.81 | 13.72 | 10.09 | 11.94 | 8.22 | 9.57 |
| GIC-H02-096 | 76 | Female | 12.9 | 14.58 | 10.42 | 12.86 | 10.7 | 9.87 |
| GIC-H02-099 | 75 | Female | 10.75 | 13.3 | 9.68 | 11.41 | 7.66 | 10.44 |
| GIC-H02-107 | 67 | Male | 14.49 | 12.34 | 10.01 | 12.22 | 5.88 | 7.82 |
| GIC-H02-108 | 66 | Male | 12.26 | 16.76 | 16.44 | 14.44 | 9.24 | 9.86 |
| GIC-H02-109 | 59 | Male | 11.79 | 15.37 | 13.24 | 19.46 | 9.69 | 10.24 |
| GIC-H02-112 | 59 | Male | 13.18 | 12.24 | 11.93 | 12.37 | 8.31 | 8.15 |
| GIC-H02-115 | 70 | Male | 12.6 | 12.5 | 12.88 | 13.41 | 7.86 | 8.21 |
| GIC-H02-117 | 73 | Male | 12.57 | 12.66 | 11.76 | 12.51 | 8.84 | 7.96 |
| GIC-H02-118 | 74 | Male | 10.65 | 11.9 | 13.37 | 11 | 10.52 | 10.97 |
| GIC-H02-123 | 47 | Male | 14.34 | 16.68 | 19.17 | 11.67 | 10.7 | 9.33 |
| GIC-H02-126 | 62 | Male | 12.55 | 13.7 | 17.5 | 17.33 | 10.47 | 10.33 |
| GIC-H02-127 | 79 | Male | 12.98 | 13.41 | 13.67 | 13.87 | 9.92 | 8.6 |
| GIC-H02-129 | 66 | Male | 13.32 | 14.68 | 15.79 | 15.21 | 11.59 | 10.23 |
| GIC-H02-133 | 69 | Female | 13.05 | 12.69 | 12.36 | 13.79 | 6.61 | 8.24 |
| GIC-H02-134 | 65 | Male | 13.9 | 14.84 | 13.83 | 14 | 8.98 | 10.26 |
| GIC-H08-2019-0239 | 63 | Male | 16.33 | 17.1 | 16.58 | 19.18 | 7.45 | 7.29 |
| GIC-H08-2019-0335 | 64 | Female | 13.4 | 19.12 | 9.91 | 15.01 | 9.2 | 10.08 |
| GIC-H08-2019-0339 | 63 | Male | 16.17 | 20.93 | 16.07 | 18.79 | 8.85 | 9.56 |
| GIC-H08-2019-0353 | 63 | Male | 16.53 | 17.02 | 15.19 | 16.84 | 10.75 | 9.18 |
| GIC-H08-2019-0391 | 46 | Female | 23.37 | 18.89 | 14.99 | 18.66 | 9.26 | 9.57 |
