# Supplementary material for: Identification of Combinations of Plasma lncRNAs and mRNAs as Potential Biomarkers for Precursor Lesions and Early Gastric Cancer
Source: J Oncol. 2022 Feb 11;2022:1458320. doi: 10.1155/2022/1458320 (PMC8856804; doi:10.1155/2022/1458320)
Supplement: Supplementary Materials — Table S1: information on lncRNA CEBPA-AS1, INHBA-AS1, AK001058, UCA1, and mRNA PPBP and RGS18. Table S2: sequences of primers used in the present study. Table S3: expression of plasma RNAs in patients with PLGC and EGC in the present study. Table S4: tumour markers in patients with PLGC and EGC in the present study. [file 1458320.f1.zip › 1458320.f1/Table S4.docx]

**Table S4: Tumour markers in patients with PLGC and EGC in the present study.**

| **Patient No.** | **Age** | **Sex** | **CEA(ng/ml)** | **AFP(ng/ml)** | **CA19-9 (U/ml)** |
| --- | --- | --- | --- | --- | --- |
| **Precursor Lesions of Gastric Cancer (PLGC)** | | | | | |
| **Gastritis** |  |  |  |  |  |
| GIC-H01-2017-0167 | 68 | Female | 0.89 | 1.78 | 8.16 |
| GIC-H01-2018-0294 | 66 | Male | 5.75 | 6.91 | 113.5 |
| GIC-H01-2018-0366 | 38 | Female | 1.08 | 4.1 | 8.08 |
| GIC-H01-2018-0446 | 41 | Female | 1.16 | 1.9 | 27.73 |
| GIC-H01-2018-0466 | 60 | Female | 1.63 | 2.59 | 8.14 |
| GIC-H01-2018-0494 | 34 | Male | 1.49 | 3 | 0.6 |
| GIC-H01-2019-0029 | 58 | Female | 1.62 | 4.08 | 4.82 |
| GIC-H01-2019-0078 | 62 | Male | 1.51 | 3.84 | 7.04 |
| GIC-H01-2019-0108 | 70 | Male | 1.39 | 2.85 | 6 |
| GIC-H01-2019-0123 | 53 | Female | 0.58 | 1.53 | 7.69 |
| **LGD & HGD** |  |  |  |  |  |
| GIC-H01-2017-0017 | 67 | Male | 2.55 | 3.85 | 11.31 |
| GIC-H01-2017-0019 | 45 | Male | 2.47 | 2.28 | 29.27 |
| GIC-H01-2017-0023 | 73 | Female | 3.91 | 7.13 | 0.6 |
| GIC-H01-2017-0029 | 45 | Male | 1.26 | 3.4 | 6.4 |
| GIC-H01-2017-0045 | 56 | Male | 2.76 | 4.16 | 6.23 |
| GIC-H01-2017-0049 | 61 | Male | 3.06 | 3.52 | 10.4 |
| GIC-H01-2017-0098 | 62 | Male | 1.14 | 2.03 | 6.17 |
| GIC-H01-2017-0100 | 43 | Male | 1.73 | 1.36 | 13.27 |
| GIC-H01-2017-0129 | 84 | Male | 7.88 | 4 | 17.98 |
| GIC-H01-2017-0136 | 50 | Male | 1.6 | 3.24 | 4.25 |
| GIC-H01-2017-0159 | 63 | Male | 2.78 | 0.68 | 6.35 |
| GIC-H01-2017-0164 | 69 | Male | 2.13 | 2.99 | 16.74 |
| GIC-H01-2017-0172 | 57 | Male | 1.34 | 1.27 | 0.6 |
| GIC-H01-2017-0195 | 66 | Male | 1.29 | 1.18 | 9.07 |
| GIC-H01-2018-0006 | 60 | Female | 1.07 | 3.51 | 15.01 |
| GIC-H01-2018-0012 | 62 | Male | 2.53 | 3.21 | 27.9 |
| GIC-H01-2018-0032 | 68 | Male | 1.82 | 2.15 | 10.47 |
| GIC-H01-2018-0045 | 72 | Male | 2.53 | 1.89 | 9.49 |
| GIC-H01-2018-0047 | 63 | Male | 2.08 | 2.37 | 0.6 |
| GIC-H01-2018-0052 | 74 | Male | 0.93 | 2.33 | 4.67 |
| GIC-H01-2018-0059 | 45 | Male | 4.07 | 5.39 | 18.34 |
| GIC-H01-2018-0068 | 63 | Male | 2.86 | 2.99 | 0.6 |
| GIC-H01-2018-0069 | 59 | Male | 1.7 | 6.2 | 5.37 |
| GIC-H01-2018-0070 | 79 | Female | 1.02 | 3.85 | 18.99 |
| GIC-H01-2018-0081 | 51 | Male | 1.8 | 3.2 | 22 |
| GIC-H01-2018-0090 | 51 | Male | 0.57 | 3.46 | 4.92 |
| GIC-H01-2018-0102 | 53 | Male | 1.15 | 2.38 | 4.24 |
| GIC-H01-2018-0103 | 79 | Female | 12.44 | 2.12 | 22.48 |
| GIC-H01-2018-0122 | 75 | Female | 3.28 | 1.91 | 5.52 |
| GIC-H01-2018-0132 | 54 | Male | 1.66 | 1.04 | 5.51 |
| GIC-H01-2018-0138 | 72 | Male | 2.71 | 8.01 | 9.78 |
| GIC-H01-2018-0157 | 65 | Male | 1.73 | 2.8 | 6.65 |
| GIC-H01-2018-0171 | 45 | Female | 0.57 | 2.38 | 5.44 |
| GIC-H01-2018-0181 | 45 | Male | 3.74 | 5.43 | 9.23 |
| GIC-H01-2018-0183 | 50 | Female | 1.32 | 2.42 | 6.64 |
| GIC-H01-2018-0189 | 56 | Male | 1.77 | 5.19 | 7.04 |
| GIC-H01-2018-0197 | 74 | Male | N/A | N/A | N/A |
| GIC-H01-2018-0203 | 50 | Male | 2.98 | 2.77 | 10.35 |
| GIC-H01-2018-0229 | 53 | Male | 1.93 | 3.4 | 6.03 |
| GIC-H01-2018-0231 | 52 | Male | 0.84 | 3.71 | 4.81 |
| GIC-H01-2018-0232 | 52 | Female | 1.5 | 3.11 | 0.6 |
| GIC-H01-2018-0251 | 47 | Female | 1.05 | 1.02 | 4.39 |
| GIC-H01-2018-0256 | 67 | Male | 7.41 | 1.41 | 15.25 |
| GIC-H01-2018-0261 | 55 | Female | 2.43 | 3.19 | 5.87 |
| GIC-H01-2018-0270 | 70 | Female | 2.99 | 2.61 | 14.12 |
| GIC-H01-2018-0301 | 57 | Male | 2.14 | 2.61 | 10.74 |
| GIC-H01-2018-0309 | 78 | Male | 2.64 | 3.72 | 15.46 |
| GIC-H01-2018-0337 | 66 | Male | 2.82 | 1.37 | 19.14 |
| GIC-H01-2018-0338 | 48 | Male | 2.11 | 2.5 | 15.93 |
| GIC-H01-2018-0346 | 51 | Female | 2.7 | 3.52 | 9.79 |
| GIC-H01-2018-0356 | 64 | Male | 4.19 | 2.66 | 13.69 |
| GIC-H01-2018-0365 | 60 | Male | 1.45 | 4.42 | 6.82 |
| GIC-H01-2018-0374 | 48 | Female | 0.97 | 2.41 | 7.54 |
| GIC-H01-2018-0382 | 64 | Male | 6.58 | 1.5 | 0.6 |
| GIC-H01-2018-0395 | 71 | Male | 1.22 | 1.64 | 7.46 |
| GIC-H01-2018-0413 | 67 | Male | 3.71 | 3.49 | 10.35 |
| GIC-H01-2018-0436 | 67 | Male | 1.56 | 2.07 | 5.24 |
| GIC-H01-2018-0448 | 61 | Male | 1.51 | 6.09 | 4.5 |
| GIC-H01-2018-0450 | 82 | Male | 2.57 | 1.29 | 20.62 |
| GIC-H01-2018-0474 | 54 | Female | 1.92 | 1.68 | 31.88 |
| GIC-H01-2018-0493 | 47 | Male | 2.25 | 1.61 | 9.33 |
| GIC-H01-2018-0495 | 63 | Male | 1.66 | 2.62 | 15.22 |
| GIC-H01-2018-0497 | 66 | Female | 1.34 | 3.17 | 9.02 |
| GIC-H01-2018-0507 | 66 | Male | 1.54 | 1.91 | 9.39 |
| GIC-H01-2018-0509 | 60 | Male | 2.36 | 0.85 | 0.6 |
| GIC-H01-2018-0513 | 67 | Male | 1.13 | 2.7 | 0.6 |
| GIC-H01-2019-0007 | 59 | Female | 1.89 | 3.7 | 22.06 |
| GIC-H01-2019-0017 | 52 | Male | 1.2 | 1.45 | 7.35 |
| GIC-H01-2019-0022 | 57 | Female | 3.52 | 1.55 | 51.48 |
| GIC-H01-2019-0075 | 55 | Male | 1.92 | 2.52 | 11.48 |
| GIC-H01-2019-0079 | 61 | Male | 1.65 | 5.34 | 6.39 |
| GIC-H01-2019-0087 | 46 | Female | 3.08 | 3.31 | 0.6 |
| GIC-H01-2019-0090 | 66 | Male | 3.74 | 2.67 | 17.22 |
| GIC-H01-2019-0095 | 62 | Male | 1.55 | 3.48 | N/A |
| GIC-H01-2019-0106 | 56 | Female | N/A | N/A | N/A |
| GIC-H01-2019-0109 | 60 | Male | 2.55 | 1.99 | 15.3 |
| GIC-H01-2019-0128 | 61 | Male | 1.34 | 3.26 | 0.6 |
| GIC-H01-2019-0133 | 63 | Male | 0.27 | 3.38 | 8.18 |
| GIC-H01-2019-0138 | 63 | Male | 3.38 | 3.55 | 12.68 |
| GIC-H02-@018A | 52 | Female | 0.99 | 1.27 | 9.99 |
| GIC-H02-@021A | 67 | Female | 1.18 | 1.15 | 9.14 |
| GIC-H02-@025A | 76 | Male | N/A | N/A | N/A |
| GIC-H02-@027A | 72 | Female | 1.6 | 1.59 | 3.17 |
| GIC-H02-@028A | 69 | Male | 0.77 | 1.28 | 1.99 |
| GIC-H02-006 | 69 | Male | N/A | N/A | N/A |
| GIC-H02-019 | 58 | Male | 7.01 | 2.17 | 11.34 |
| GIC-H02-030 | 76 | Male | 2.96 | 1.33 | 25.05 |
| GIC-H02-043 | 74 | Male | 2.58 | 2.66 | 6.64 |
| GIC-H02-044 | 70 | Male | N/A | N/A | N/A |
| GIC-H02-057 | 86 | Male | N/A | N/A | N/A |
| GIC-H02-060 | 61 | Male | 3.06 | 2.48 | 8.52 |
| GIC-H02-073 | 68 | Male | N/A | N/A | N/A |
| GIC-H02-080 | 42 | Male | 2.48 | 2.36 | 4.27 |
| GIC-H02-086 | 48 | Male | 2.5 | 2.78 | 9.91 |
| GIC-H02-091 | 62 | Male | 2.43 | 0.61 | 0.6 |
| GIC-H02-095 | 71 | Male | 3.03 | 19.53 | 44.83 |
| GIC-H02-100 | 49 | Male | 2.76 | 2.29 | 7.16 |
| GIC-H02-111 | 63 | Male | 6.42 | 2.94 | 18.1 |
| GIC-H02-119 | 63 | Male | 3.49 | 3.3 | 8.56 |
| GIC-H02-120 | 69 | Male | 2.57 | 2.63 | 11.89 |
| GIC-H02-128 | 49 | Male | N/A | N/A | N/A |
| GIC-H02-131 | 63 | Male | 2.02 | 7.72 | 40.1 |
| GIC-H02-136 | 56 | Male | 1.74 | 1.42 | 8.23 |
| GIC-H02-137 | 55 | Male | 1.55 | 2.54 | 23.88 |
| GIC-H08-2018-0157 | 56 | Female | 1.7 | 1.7 | 9.5 |
| GIC-H08-2019-0159 | 47 | Male | 3.2 | 5.1 | 18.4 |
| GIC-H08-2019-0349 | 46 | Male | 3.7 | 2.2 | 13.7 |
| GIC-H08-2019-0399 | 67 | Female | 1.3 | 2.8 | 6.2 |
| GIC-H08-2019-0415 | 72 | Male | 1.7 | 2.8 | 4.6 |
| **Early Gastric Cancer (EGC)** | | | | | |
| GIC-H01-2017-0014 | 61 | Male | 1.93 | 2.38 | 9.03 |
| GIC-H01-2017-0036 | 74 | Male | 5.38 | 1.95 | 8.58 |
| GIC-H01-2017-0051 | 67 | Male | N/A | N/A | N/A |
| GIC-H01-2017-0081 | 51 | Male | 1.52 | 1.25 | 5.85 |
| GIC-H01-2017-0082 | 67 | Male | 1.74 | 2.19 | 8.02 |
| GIC-H01-2017-0096 | 64 | Male | 2.59 | 3.42 | 5.82 |
| GIC-H01-2017-0117 | 66 | Male | 1.53 | 3.56 | N/A |
| GIC-H01-2017-0130 | 50 | Female | 0.74 | 1.33 | 22.5 |
| GIC-H01-2017-0197 | 50 | Male | 5.02 | 7.41 | 15.02 |
| GIC-H01-2017-0211 | 51 | Female | N/A | N/A | N/A |
| GIC-H01-2018-0001 | 65 | Male | 2.2 | 2.23 | 7.45 |
| GIC-H01-2018-0024 | 48 | Male | 1.57 | 1.81 | 13.13 |
| GIC-H01-2018-0029 | 72 | Male | 2.56 | 4.43 | 14.65 |
| GIC-H01-2018-0034 | 45 | Male | 1.93 | 2.28 | 8.55 |
| GIC-H01-2018-0082 | 63 | Male | 5.22 | 1.13 | 18.7 |
| GIC-H01-2018-0083 | 35 | Male | 0.76 | 2.59 | 10.05 |
| GIC-H01-2018-0131 | 72 | Male | 2.33 | 5.84 | 5.97 |
| GIC-H01-2018-0159 | 62 | Female | 0.8 | 12.82 | 8.94 |
| GIC-H01-2018-0165 | 75 | Female | 4.62 | 2.31 | 12.92 |
| GIC-H01-2018-0169 | 63 | Female | 1.28 | 3.18 | 8.24 |
| GIC-H01-2018-0188 | 62 | Male | N/A | N/A | N/A |
| GIC-H01-2018-0196 | 43 | Female | 1.42 | 4.89 | 8.78 |
| GIC-H01-2018-0207 | 64 | Male | 1.41 | 2.11 | 5.37 |
| GIC-H01-2018-0230 | 48 | Male | 1.16 | 2.1 | 4.72 |
| GIC-H01-2018-0233 | 58 | Male | 1.33 | 2.83 | 7.54 |
| GIC-H01-2018-0239 | 58 | Male | 5.23 | 3.66 | 8.5 |
| GIC-H01-2018-0241 | 63 | Female | 2.68 | 2.36 | 7.52 |
| GIC-H01-2018-0249 | 57 | Male | 1.38 | 2.73 | 13.34 |
| GIC-H01-2018-0250 | 60 | Female | 1.36 | 6.8 | 6.9 |
| GIC-H01-2018-0295 | 57 | Female | 1.7 | 3.07 | 24.21 |
| GIC-H01-2018-0308 | 44 | Male | 1.59 | 2.74 | 9.53 |
| GIC-H01-2018-0313 | 59 | Male | N/A | N/A | N/A |
| GIC-H01-2018-0324 | 70 | Male | 4.97 | 1.31 | 10.44 |
| GIC-H01-2018-0343 | 48 | Male | 1.37 | 2.06 | 7.34 |
| GIC-H01-2018-0353 | 71 | Female | 2.95 | 6.57 | 17.83 |
| GIC-H01-2018-0373 | 73 | Male | 3.78 | 1.6 | 15.36 |
| GIC-H01-2018-0377 | 78 | Male | 6.32 | 1.44 | 18.74 |
| GIC-H01-2018-0406 | 78 | Male | 1.55 | 2.36 | 8.24 |
| GIC-H01-2018-0411 | 39 | Male | 1.18 | 2.52 | 5.92 |
| GIC-H01-2018-0414 | 74 | Male | 1.41 | 2.43 | 12.57 |
| GIC-H01-2018-0420 | 59 | Female | 1.66 | 2.84 | 2.9 |
| GIC-H01-2018-0428 | 60 | Male | 2.3 | 2.19 | 14 |
| GIC-H01-2018-0445 | 70 | Male | 6.51 | 2.17 | 28.56 |
| GIC-H01-2018-0473 | 54 | Male | 2.26 | 4.06 | 0.6 |
| GIC-H01-2018-0477 | 64 | Male | N/A | N/A | N/A |
| GIC-H01-2018-0514 | 60 | Male | 0.97 | 3.48 | 4.91 |
| GIC-H01-2019-0012 | 57 | Male | 2.62 | 3.06 | 9.58 |
| GIC-H01-2019-0013 | 54 | Male | 4.67 | 2.36 | 29.41 |
| GIC-H01-2019-0016 | 55 | Male | 2.89 | 2.96 | 7.91 |
| GIC-H01-2019-0018 | 64 | Male | 3.1 | 1.75 | 13.68 |
| GIC-H01-2019-0020 | 61 | Male | 2.76 | 1.5 | 10.02 |
| GIC-H01-2019-0026 | 69 | Male | N/A | N/A | N/A |
| GIC-H01-2019-0028 | 70 | Male | 3.5 | 1.52 | 22.58 |
| GIC-H01-2019-0040 | 70 | Male | 2.83 | 1.71 | 24.52 |
| GIC-H01-2019-0041 | 72 | Male | N/A | N/A | N/A |
| GIC-H01-2019-0044 | 48 | Male | 1.46 | 2.94 | 11.82 |
| GIC-H01-2019-0054 | 72 | Male | 2.85 | 2.69 | 16.18 |
| GIC-H01-2019-0055 | 55 | Male | 0.74 | 2.16 | 7.76 |
| GIC-H01-2019-0056 | 55 | Male | 1.72 | 2.95 | 7.58 |
| GIC-H01-2019-0061 | 77 | Male | 5.4 | 2.85 | 10.65 |
| GIC-H01-2019-0064 | 74 | Female | 2.65 | 2.44 | 14.58 |
| GIC-H01-2019-0069 | 67 | Male | 1.63 | 1.72 | 12.08 |
| GIC-H01-2019-0072 | 52 | Male | 0.57 | 1.71 | 4.21 |
| GIC-H01-2019-0085 | 70 | Female | N/A | N/A | N/A |
| GIC-H01-2019-0115 | 58 | Male | 1.2 | 1.98 | 0.6 |
| GIC-H01-2019-0118 | 69 | Male | 1.88 | N/A | 3.9 |
| GIC-H01-2019-0122 | 62 | Male | 2.4 | 2.28 | 39.81 |
| GIC-H01-2019-0124 | 74 | Female | 0.2 | 1.99 | 12.14 |
| GIC-H01-2019-0136 | 70 | Female | 4.36 | 2.12 | 11.31 |
| GIC-H01-2019-0139 | 64 | Male | 1.74 | 4.4 | 6.45 |
| GIC-H01-2019-0158 | 69 | Female | 1.2 | 4.07 | 10.4 |
| GIC-H01-2019-0162 | 54 | Male | N/A | N/A | N/A |
| GIC-H02-@001A | 70 | Male | 3.68 | N/A | 24.95 |
| GIC-H02-@009A | 64 | Male | 2.46 | 2.45 | 6.26 |
| GIC-H02-@012A | 62 | Male | 2.58 | 1.45 | 0.6 |
| GIC-H02-@014A | 64 | Male | 2.35 | 2.14 | 6.56 |
| GIC-H02-@017A | 72 | Male | 2.31 | 2.82 | 11.63 |
| GIC-H02-@022A | 75 | Male | 1.08 | 1.9 | 6.52 |
| GIC-H02-@024A | 62 | Male | 1.42 | 2.74 | 6.88 |
| GIC-H02-@026A | 64 | Male | 0.69 | 2.69 | 6.17 |
| GIC-H02-007 | 71 | Male | 1.21 | 3.57 | 10.9 |
| GIC-H02-008 | 60 | Male | 5.7 | 1.99 | 9.69 |
| GIC-H02-020 | 50 | Male | 4.1 | 4.56 | 13.81 |
| GIC-H02-021 | 53 | Male | 1.5 | 1.74 | 5.31 |
| GIC-H02-022 | 67 | Male | 2.48 | 2.23 | 17.73 |
| GIC-H02-031 | 55 | Male | 6.45 | 2.85 | 0.6 |
| GIC-H02-040 | 58 | Male | 1.77 | 2.05 | 3.46 |
| GIC-H02-041 | 54 | Male | N/A | N/A | N/A |
| GIC-H02-078 | 61 | Male | 1.29 | 1.74 | 8.53 |
| GIC-H02-085 | 79 | Female | 4.13 | 0.62 | 26.98 |
| GIC-H02-087 | 52 | Male | 1.23 | N/A | N/A |
| GIC-H02-088 | 73 | Female | 1.33 | 1.79 | 10.08 |
| GIC-H02-090 | 58 | Male | 3.1 | 3.28 | 5.53 |
| GIC-H02-093 | 61 | Male | 4.38 | 2.21 | 11.47 |
| GIC-H02-094 | 51 | Male | 3.13 | 5.24 | 18.84 |
| GIC-H02-096 | 76 | Female | 2.88 | 2.46 | 11.64 |
| GIC-H02-099 | 75 | Female | 1.26 | 1.06 | 10.26 |
| GIC-H02-104 | 64 | Male | 1.7 | 2.76 | 33.05 |
| GIC-H02-107 | 67 | Male | N/A | N/A | N/A |
| GIC-H02-108 | 66 | Male | 3.49 | 5.19 | N/A |
| GIC-H02-109 | 59 | Male | N/A | N/A | N/A |
| GIC-H02-112 | 59 | Male | N/A | N/A | N/A |
| GIC-H02-115 | 70 | Male | 1.8 | 1.39 | 10.37 |
| GIC-H02-116 | 79 | Female | 5.19 | 1.2 | 37.36 |
| GIC-H02-117 | 73 | Male | N/A | N/A | N/A |
| GIC-H02-118 | 74 | Male | 2.82 | 3.02 | 13.71 |
| GIC-H02-121 | 61 | Female | 1.01 | 1.34 | 11.49 |
| GIC-H02-123 | 47 | Male | 2.51 | 3.43 | 21.38 |
| GIC-H02-126 | 62 | Male | 4.66 | 1.82 | 16.95 |
| GIC-H02-127 | 79 | Male | 2.96 | 5.83 | 11.47 |
| GIC-H02-129 | 66 | Male | 1.29 | 2.19 | 11.54 |
| GIC-H02-133 | 69 | Female | 2.54 | 3.78 | 29.24 |
| GIC-H02-134 | 65 | Male | 1.19 | 2.4 | 6.46 |
| GIC-H08-2019-0165 | 44 | Female | 4.2 | 3.1 | 119.8 |
| GIC-H08-2019-0177 | 58 | Female | 0.4 | 0.5 | 11.5 |
| GIC-H08-2019-0185 | 53 | Female | 1.1 | 3.1 | 0.8 |
| GIC-H08-2019-0187 | 59 | Male | 10.6 | 0.7 | ＞1000 |
| GIC-H08-2019-0203 | 51 | Male | 4.2 | 12.1 | 13.9 |
| GIC-H08-2019-0207 | 62 | Male | 2.3 | 1.7 | 20.3 |
| GIC-H08-2019-0231 | 56 | Female | 1.8 | 3.5 | 1.2 |
| GIC-H08-2019-0239 | 63 | Male | 2.7 | 2.7 | 5.2 |
| GIC-H08-2019-0255 | 62 | Male | 40.89 | 1.17 | 0.8 |
| GIC-H08-2019-0257 | 67 | Female | 4.5 | 4.3 | 35.5 |
| GIC-H08-2019-0267 | 80 | Male | 1.9 | ＜0.6 | 119 |
| GIC-H08-2019-0273 | 74 | Male | 2.6 | 1.5 | 45.2 |
| GIC-H08-2019-0275 | 61 | Male | 2.5 | 1.4 | 8.5 |
| GIC-H08-2019-0291 | 49 | Female | 1.55 | 3.46 | 11.2 |
| GIC-H08-2019-0299 | 57 | Male | 6.3 | 6 | 5.3 |
| GIC-H08-2019-0303 | 67 | Male | 2.7 | 1 | 5.6 |
| GIC-H08-2019-0325 | 62 | Female | 1.48 | 1.24 | 5.7 |
| GIC-H08-2019-0335 | 64 | Female | 1.5 | 2.5 | 8.6 |
| GIC-H08-2019-0339 | 63 | Male | 2.11 | 3.13 | 6.9 |
| GIC-H08-2019-0341 | 75 | Male | 3.6 | 2.9 | 11.4 |
| GIC-H08-2019-0351 | 51 | Male | 2.7 | 1.4 | 10.5 |
| GIC-H08-2019-0353 | 63 | Male | 6 | 1.7 | 5.5 |
| GIC-H08-2019-0357 | 48 | Male | 2.2 | 2.3 | 2.2 |
| GIC-H08-2019-0359 | 57 | Female | 4.7 | 1.8 | 30.8 |
| GIC-H08-2019-0363 | 62 | Male | 5.6 | 6.5 | 7.3 |
| GIC-H08-2019-0373 | 50 | Male | 3.8 | 1.4 | 7.1 |
| GIC-H08-2019-0375 | 50 | Male | 6.9 | 4.7 | 20.1 |
| GIC-H08-2019-0379 | 51 | Male | 1.6 | 1.2 | 9.8 |
| GIC-H08-2019-0391 | 46 | Female | 0.3 | 4.5 | 12.2 |
| GIC-H08-2019-0401 | 52 | Male | 3.3 | 3.7 | 4.1 |
